# Hydroxyl Radical Production by Air Pollutants in Epithelial Lining Fluid Governed by Interconversion and Scavenging of Reactive Oxygen Species

**DOI:** 10.1021/acs.est.1c03875

**Published:** 2021-10-05

**Authors:** Steven Lelieveld, Jake Wilson, Eleni Dovrou, Ashmi Mishra, Pascale S. J. Lakey, Manabu Shiraiwa, Ulrich Pöschl, Thomas Berkemeier

**Affiliations:** † Multiphase Chemistry Department, Max Planck Institute for Chemistry, 55128 Mainz, Germany; ‡ Department of Chemistry, University of California, Irvine, Irvine, California 92697, United States

**Keywords:** reactive oxygen species, PM2.5, epithelial lining fluid, oxidative stress

## Abstract

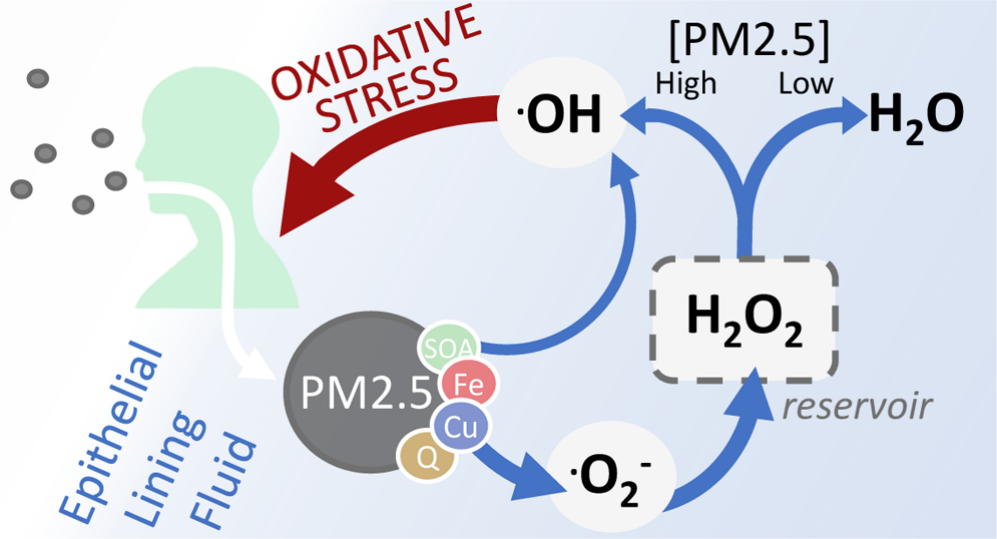

Air pollution is a major risk factor for human health. Chemical reactions in the epithelial lining fluid (ELF) of the human respiratory tract result in the formation of reactive oxygen species (ROS), which can lead to oxidative stress and adverse health effects. We use kinetic modeling to quantify the effects of fine particulate matter (PM2.5), ozone (O_3_), and nitrogen dioxide (NO_2_) on ROS formation, interconversion, and reactivity, and discuss different chemical metrics for oxidative stress, such as cumulative production of ROS and hydrogen peroxide (H_2_O_2_) to hydroxyl radical (OH) conversion. All three air pollutants produce ROS that accumulate in the ELF as H_2_O_2_, which serves as reservoir for radical species. At low PM2.5 concentrations (<10 μg m^–3^), we find that less than 4% of all produced H_2_O_2_ is converted into highly reactive OH, while the rest is intercepted by antioxidants and enzymes that serve as ROS buffering agents. At elevated PM2.5 concentrations (>10 μg m^–3^), however, Fenton chemistry overwhelms the ROS buffering effect and leads to a tipping point in H_2_O_2_ fate, causing a strong nonlinear increase in OH production. This shift in ROS chemistry and the enhanced OH production provide a tentative mechanistic explanation for how the inhalation of PM2.5 induces oxidative stress and adverse health effects.

## Abstract 2

This study uses kinetic modeling to provide quantitative and mechanistic insights into the formation of reactive oxygen species (ROS) in the lung after inhalation of the air pollutants PM2.5, NO_2_, and O_3_.

## Introduction

Ambient air pollution is responsible for 4–9 million excess deaths per year.^1−3^ Air pollutants can cause and exacerbate ischemic heart disease (e.g., myocardial infarction), cerebrovascular disease (e.g., stroke), lower respiratory infections (e.g., pneumonia), and chronic obstructive pulmonary disease (COPD).^4−6^ The air pollutants that most strongly correlate with negative health outcomes are nitrogen dioxide (NO_2_), ozone (O_3_), and fine particulate matter with a diameter less than 2.5 μm (PM2.5), with the latter likely contributing more than 80% to the total excess mortality.^7,8^

PM2.5 is a complex mixture that can encompass thousands of different chemical constituents, each having distinct properties. PM2.5 originates from both natural and anthropogenic sources, including mineral dust from deserts, gasoline and diesel motor exhausts, tire and brake wear, power generation, residential energy use, agriculture, biomass burning, cooking, and cigarette smoking. Because of the great heterogeneity in both PM2.5 composition and sources, targeted PM2.5 pollution control is challenging, and, to date, there is no clear connection between one particular PM2.5 constituent and mortality estimates.^9−12^ In spite of fundamental challenges studying causal relationships between air pollutants and health outcomes, it has been generally accepted that the underlying pathology of air pollutant exposure includes oxidative stress and systemic inflammation.^7,13−15^ Moreover, in recent years, the oxidative potential of PM2.5 has become a common metric for measuring PM2.5 toxicity.^13,16−19^ The oxidative potential of PM2.5 has been shown to vary greatly among sampling sites and proximity to the emitting source.^16,20−22^ Based on case-crossover studies, it has been suggested that the risk of respiratory illness and myocardial infarction was increased in exposure episodes with high PM2.5 oxidative potential.^13,23^

PM2.5 contains redox-active components, most notably copper, iron, secondary organic aerosols (SOA), and quinones, which trigger the formation of reactive oxygen species (ROS) in the epithelial lining fluid (ELF) of the respiratory tract.^14,24−28^ The umbrella term “ROS” encompasses several highly reactive molecules, including hydrogen peroxide (H_2_O_2_), the hydroperoxyl radical (HO_2_), the superoxide radical anion (O_2_^–^), and the hydroxyl radical (OH).^29^ Their reactivity and stability vary greatly, with H_2_O_2_ being the most stable, and OH the most reactive.^30^ ROS may induce oxidative stress and inflammation in the ELF, thereby causing adverse health effects.^14,24,31−33^

NO_2_ is an irritant gas that has been linked to mortality in epidemiological studies.^34,35^ However, because NO_2_ is often co-emitted with PM2.5 and other pollutants in combustion processes, it remains unclear if it poses an independent health risk.^36,37^ In the ELF, NO_2_ can consume antioxidants and form nitrite (NO_2_^–^) in the process.^38,39^ The oxidized forms of antioxidants are typically nontoxic, but their reactive intermediates have been suggested to form ROS in small yields in the case of the glutathiyl radical.^39,40^

Exposure to O_3_ has been shown to exacerbate asthma and increase respiratory and circulatory mortality.^8,41,42^ It is known to react with alkenes by addition to the C–C double bond, leading to lipid peroxidation and forming a variety of oxidized reaction products, including Criegee intermediates and hydroperoxides.^43−45^ However, while the chemical properties of NO_2_ and O_3_ are well understood, the mechanisms behind their health effects and contribution to ROS formation in the ELF, remain unclear.

In cells, several mechanisms prevent the formation of ROS, or intercept these highly reactive molecules before causing oxidative stress.^30^ The interception of ROS includes chemical reactions leading to unreactive products (ROS scavenging) and chemical conversion into less reactive ROS.^30,46^ In the ELF, this task is fulfilled by low-molecular-mass antioxidants and antioxidant enzymes.^14,24,47^ The enzyme superoxide dismutase (SOD) efficiently shuttles O_2_^–^ into the less reactive H_2_O_2_, whereas the enzyme catalase is the major natural sink of H_2_O_2_ in the ELF.^48^ Together, these endogenous processes lead to a ROS buffering effect that helps to maintain physiological ROS concentrations and prevents the formation of highly reactive and noxious OH radicals.^49^ Oxidative stress commonly refers to the imbalance between these natural defense mechanisms and ROS production, leading to an excess of ROS.^50,51^

Previously, the kinetic model KM-SUB-ELF was developed and applied to calculate the chemical exposure-response relationship between PM2.5 and ROS concentrations in the ELF.^13,14,28^ The model showed that momentary ROS concentrations can exceed concentrations characteristic for healthy humans (100 nmol L^–1^) after exposure to PM2.5; furthermore, important redox-active air pollutants as well as cyclic reaction mechanisms with endogenous reaction partners were identified.^14^ Recent epidemiological studies with air monitoring and KM-SUB-ELF modeling have found positive associations between long-term exposure to iron, copper, and ROS with the risks of respiratory and cardiovascular diseases.^52−54^ A significant positive association was also observed between ROS levels in ELF and COVID-19 incidence.^55^

The metric of momentary ROS concentration is dominated by chemical species with relatively long lifetimes, such as H_2_O_2_, and foregoes short-lived species like OH that are known to cause damage and oxidative stress.^56,57^ While the production mechanisms of H_2_O_2_ and OH in ELF are closely connected, their yields and concentrations may not. This becomes pertinent, for example, in the presence of transition-metal ions, where Fenton chemistry causes OH formation through decomposition of H_2_O_2_.^58,59^ While the momentary ROS concentration decreases through Fenton chemistry, the overall ROS reactivity and potential to induce oxidative stress may strongly increase.^30^ Thus, chemical metrics for oxidative stress are needed that take into account not only the quantity but also the chemical identities of produced ROS.

In this study, the kinetic model KM-SUB-ELF is comprehensively extended and embedded into a new framework for analysis of model output that enables novel insights on the production, interconversion, and scavenging of ROS. The most notable extensions to KM-SUB-ELF are the expansion of the biological antioxidant system by explicit inclusion of the ROS buffering enzymes catalase and SOD, the inclusion of the air pollutant NO_2_, and revision of uptake and chemistry of the pollutant O_3_. With these additions, the model is now able to capture the fundamental competition between the antioxidant system and the mixture of air pollutants. A new and comprehensive chemical source apportionment pinpoints the chemical species that are most important for production, interconversion, and scavenging of ROS in different pollution scenarios. Based on these learnings, we propose the new chemical metrics of cumulative ROS production rate and H_2_O_2_-to-OH conversion fraction to represent the potential of air pollution to induce oxidative stress.

## Methods

The kinetic model presented in this study builds on the previously published model KM-SUB-ELF,^14^ which is based on the kinetic multilayer model for aerosol surface and bulk chemistry (KM-SUB).^60^ KM-SUB-ELF consists of three compartments, the lung gas phase, the surfactant layer of the ELF, and the bulk ELF. The model explicitly treats airflow into and out of the lung, adsorption of gases onto the ELF’s surfactant layer, desorption from the surfactant layer, surface-bulk exchange between surfactant layer and bulk ELF, bulk diffusion within the ELF, as well as chemical reactions in the gas and aqueous phases. The temporal evolution of reactants is calculated by solving a system of ordinary differential equations. Table S1 outlines the chemical reactions treated in KM-SUB-ELF, including 23 gas-phase reactions, six reactions in the surfactant layer, and 96 aqueous-phase reactions in the bulk ELF. Rate coefficients for the gas-phase chemical reactions of H_2_O_2_, HO_2_, NO, NO_2_, and O_3_ are adopted from the Master Chemical Mechanism (MCM).^61,62^ The aqueous-phase redox chemistry in the model was validated previously against experimental studies on H_2_O_2_ and OH formation in surrogate ELF.^14,24,25^ H_2_O_2_ and OH production from SOA are parameterized based on experimental observations.^14,27,63^ The ELF is subdivided into six different layers, one surfactant layer containing lipids (1-palmitoyl-2-oleoylglycerol, POG) and a surfactant protein (SP-B), and five bulk layers containing four antioxidants (detailed in the Supporting Information, Section S1) and two antioxidant enzymes (detailed in Section S2). Moreover, a first-order loss reaction of OH is included to account for OH reacting with organic matter that is present in the ELF (Supporting Information, Section S3). Particulate pollutant concentrations in the ELF are derived as described previously (Supporting Information, Section S4).^14^ In short, ambient PM2.5 from a 2 h exposure window is deposited into the lung with a deposition fraction of 0.45.^64^ Mass fractions of the redox-active constituents in PM2.5 are obtained from a range of field measurements (Tables S5–S7).^14^ Because NO_2_ and PM2.5 are often co-emitted, in our calculations the gas-phase concentration of NO_2_ is co-varied with PM2.5 concentration with a factor of 1 μg m^–3^ NO_2_ for each μg m^–3^ PM2.5.^65^ Due to the more complex relationship of O_3_ and PM2.5, O_3_ is treated with a constant concentration of 30 μg m^–3^ (corresponding to ∼15 ppb at 1 atm, 298 K), irrespective of other pollutant concentrations, to resemble an atmospheric background concentration.^66,67^ In Figure 4, three distinct pollutant exposure scenarios are highlighted that have the following characteristics: (1) “clean”, with concentrations of 5 μg m^–3^ PM2.5, 5 μg m^–3^ NO_2_, and 20 μg m^–3^ O_3_; (2) “urban”, with 30, 30, and 60 μg m^–3^; and (3) “megacity”, with 300, 300, and 60 μg m^–3^ for the same pollutants, respectively. Gas exchange between the lungs and the ambient air is included to simulate breathing (detailed in the Supporting Information, Section S5). Volatile vapors partition to the ELF according to Henry’s law. Acids and conjugate bases are assumed to maintain equilibrium and the position of the acid–base equilibria is determined using the pKa of the species involved and a pH of 7 (detailed in the Supporting Information, Section S6). A full list of input parameters as used in KM-SUB-ELF is presented in Table S2. The definitions and equations for the calculation of the chemical metrics for oxidative stress are presented in Tables S3 and S4, respectively. To facilitate discussion, we establish a standardized composition of PM2.5, representing median mass fractions of the redox-active PM2.5 constituents copper and iron ions, quinones, and SOA, as determined in field measurements (Tables S5–S7). In figures, standard PM2.5 composition is indicated with solid lines, whereas markers will indicate simulation results using explicit composition data.

## Results and Discussion

**Figure 1:**
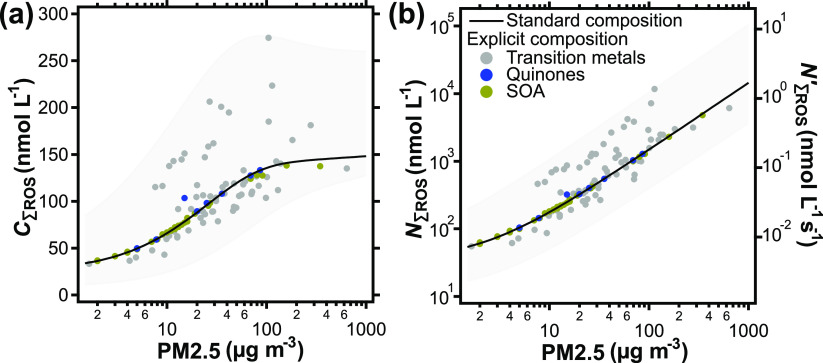
(a) Total ROS concentration, *C*_∑ROS_, and (b) cumulative production of ROS, *N*_∑ROS_, in the ELF as a function of ambient PM2.5 concentration after a 2 h period of pollutant exposure. The right axis in (b) shows the cumulative ROS production rate (*N’*_∑ROS_, Table S4). The solid lines represent a standard PM2.5 composition based on median mass fractions of redox-active constituents of 0.03% copper, 0.8% iron, 0.002% quinones, and 33% SOA. Markers represent explicit PM2.5 composition field data for the indicated redox-active constituents (Tables S5–S7) to illustrate the sensitivity and variance induced by the PM2.5 constituents. Shadings indicate a dynamic range of each concentration metric as a function of PM2.5 composition and concentration of gaseous pollutants.

Exposure to PM2.5, NO_2_, and O_3_ results in ROS formation in the ELF. Figure 1a shows the total ROS concentration in the ELF at the end of 2 h of pollutant exposure, *C*_∑ROS_, computed using KM-SUB-ELF for a range of pollutant concentrations and for different PM2.5 compositions. We use “∑ROS” to indicate the sum of all ROS treated explicitly in this study, i.e., H_2_O_2_, O_2_^–^, HO_2_, and OH. The color-coded markers in Figure 1a represent simulation results using mass fractions of single PM2.5 redox-active constituent classes (gray: transition metals, yellow: SOA, blue: quinones) that were obtained in field measurements (Tables S5–S7).^14^ In each calculation, the mass fractions of the other constituent classes are kept at their median mass fraction. The median mass fractions of copper and iron ions, quinones, and SOA are determined to be 0.03, 0.8, 0.002, and 33%, respectively (Tables S5–S7). To illustrate the variability in PM2.5 composition, the individual mass fractions obtained from field data are presented in Figure S1. To illustrate the variability in PM2.5 composition, the individual mass fractions obtained from field data are presented in Figure S1.

The black solid line in Figure 1a represents the total momentary ROS concentration, *C*_∑ROS_, that results from the standard PM2.5 composition using median mass fractions of all redox-active constituents. The variance pattern of markers around the line in Figure 1a indicates that the transition-metal-ion mass fractions dominate the influence of PM2.5 composition on model output. This is due to both, a strong model sensitivity, and a large variability of transition-metal mass fractions obtained in field measurements (Figure S1). However, the overall model behavior is well represented by the line representing a standardized PM2.5 composition. In the following, we will use this standard composition to assess the effect of ambient PM2.5 concentration on model results. For the purpose of discussion in this study, we categorize pollution levels according to the PM2.5 concentrations as “low” (<10 μg m^–3^ PM2.5), “typical urban” (10–100 μg m^–3^ PM2.5), and “very high” (>100 μg m^–3^ PM2.5) pollution. The model predicts that *C*_∑ROS_ ranges from ∼30 nmol L^–1^ at low pollution levels to over 250 nmol L^–1^ at very high pollution. *C*_∑ROS_ induced by typical urban exposure is found to range between ∼70 and ∼250 nmol L^–1^, which is consistent with ROS concentrations measured in exhaled breath condensate of humans.^68,69^ In Figure S2, *C*_∑ROS_ after 2 h of exposure is compared to the arithmetic mean of *C*_∑ROS_ during these 2 h. Qualitatively, both metrics for momentary ROS concentration show very similar behavior, but the time average exhibits overall slightly lower values due to the initial increase in ROS concentrations.

We note that, to the knowledge of the authors, rates of antioxidant replenishment in ELF have not been reported previously. Kelly et al. showed experimentally that antioxidants do not fully deplete in the ELF of healthy volunteers upon exposure to NO_2_.^70^ A partial depletion of antioxidants does not affect modeling results (Figure S3) as reactions with the oxidized forms of transition-metal ions and quinones are fast and do not represent a bottleneck for redox cycling in the ELF. Thus, for simplicity, antioxidant replenishment is considered sufficiently fast within the 2 h exposure window and antioxidant concentrations are kept constant in the model calculations. Otherwise, without replenishment of antioxidants, exposure to air pollution with NO_2_ concentrations above 100 μg m^–3^ (corresponding to ∼50 ppb) leads to a spike in *C*_∑ROS_ (Figure S3a), caused by full depletion of antioxidants within the 2 h exposure time (Figure S3b). This model result provides evidence for a higher susceptibility to air pollution at critically low antioxidant levels.

Figure 1b introduces the metric of cumulative production of ROS, *N*_∑ROS_, as an additional chemical endpoint for the effects of air pollution on human health.
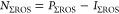
Here, *P*_∑ROS_ is the gross chemical production of ROS (in nmol L^–1^), i.e., the time-integrated sum of all chemical production terms within the 2 h of exposure. *I*_∑ROS_ (in nmol L^–1^) is the time-integrated sum of ROS molecules that originate from interconversion between individual ROS, and is subtracted to avoid double counting of ROS. Following the solid line of standard PM2.5 composition, *N*_∑ROS_ is found to increase linearly with air pollution exposure and ranges from less than 100 nmol L^–1^ at low concentrations of air pollutants, to over 1 μmol L^–1^ at very high concentrations. While the metric *C*_∑ROS_ is a measure for stable ROS in the ELF, *N*_∑ROS_ accounts for all ROS produced, irrespective of reactivity or lifetime.

**Figure 2:**
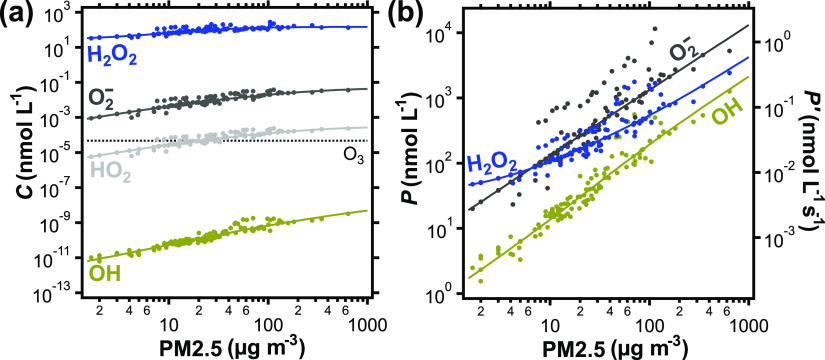
(a) Individual ROS concentrations, *C*, and (b) gross chemical production, *P*, of individual ROS in the ELF as a function of ambient PM2.5 concentration. The right axis in (b) shows the gross chemical production rate of individual ROS (*P*′, Table S4). The solid lines represent a standard PM2.5 composition, and the markers represent explicit PM2.5 compositions derived from field data (Tables S5–S7). *C*_O2−_ and *C*_HO2_ in (a) are calculated using acid–base equilibria, as detailed in the Supporting Information, Section S6. In (b), *P*_O2−_ also includes *P*_HO2_. The dotted line in (a) shows the steady-state O_3_ concentration in the ELF.

Figure 2a shows the contributions of individual species to the total momentary ROS concentration in the ELF. *C*_∑ROS_ is found to be dominated by the H_2_O_2_ concentration, *C*_H2O2_, with *C*_O2−_, *C*_HO2_, and *C*_OH_ having only negligible contributions.^14^ Due to the small contribution of HO_2_ to *C*_∑ROS_ at pH 7 and the fast interconversion between both species, we use O_2_^–^ for the sum of the HO_2_/O_2_^–^ acid–base pair in the following discussions for simplicity. Note that the high and unspecific reactivity of OH with all organic matter in the ELF leads to uncertainty in *C*_OH_, which is further detailed in the Supporting Information, Section S3.

Figure 2b outlines the gross chemical production, *P*, of individual ROS in the ELF, which is calculated by time integrating all production terms of the individual species. Over most of the investigated PM2.5 concentration range, O_2_^–^ shows the largest production (10 nmol L^–1^ to 10 μmol L^–1^), followed by H_2_O_2_ (40 nmol L^–1^ to 4 μmol L^–1^), and OH (1 nmol L^–1^ to 2 μmol L^–1^). These results are consistent with Gonzalez et al.,^71^ who found OH production in the range of 0.5–1.5 μmol L^–1^ after 2 h incubation of 1 μmol L^–1^ iron (corresponding to ∼500 μg m^–3^ PM2.5) in bronchoalveolar lavage fluid.

Figure S4 breaks down the contributions of the individual pollutants PM2.5, NO_2_, and O_3_ to the gross chemical productions of ROS shown in Figure 2b. Production of O_2_^–^ and OH can be largely attributed to PM2.5 (Figure S4a), whereas H_2_O_2_ is produced in significant quantity from O_3_ reacting with unsaturated lipids in the surfactant layer (R27, Table S1 and Figure S4b). Thus, H_2_O_2_ dominates gross chemical production of ROS at low ambient pollutant concentrations because of the constant O_3_ background concentration, which is assigned irrespective of PM2.5 and NO_2_ levels. Accordingly, at a PM2.5 concentration of 7 μg m^–3^, production of O_2_^–^ surpasses production of H_2_O_2_. We note that, to the knowledge of the authors, H_2_O_2_ yield from surfactant ozonolysis in the ELF has not been reported previously. H_2_O_2_ yields from gas-phase ozonolysis of small alkenes in the presence of bulk water are found between 3 and 24%.^43^ A compound that closely resembles fatty acid residues in mono-unsaturated lipids is methyl oleate, which showed an H_2_O_2_ yield of 17% and was taken as a reference for this study.^72^ The exact yield of H_2_O_2_ will also depend on the water content in the lipid layer of the ELF, which determines Criegee intermediate fate, and warrants further investigation.^43,72^

Compared to momentary concentrations of individual ROS, which span 10–12 orders of magnitude (Figure 2a), the individual gross chemical productions are within about 2 orders of magnitude from each other at low pollutant concentrations, and within about 1 order of magnitude at higher pollutant concentrations (Figure 2b). This finding can be attributed to the chain of ROS interconversions in the ELF: O_2_^–^ is often produced initially and then successively converted into H_2_O_2_ and OH. The decreasing disparity between individual production terms with increasing PM2.5 concentration in Figure 2b suggests that ROS are interconverted more efficiently at higher pollution levels.

**Figure 3:**
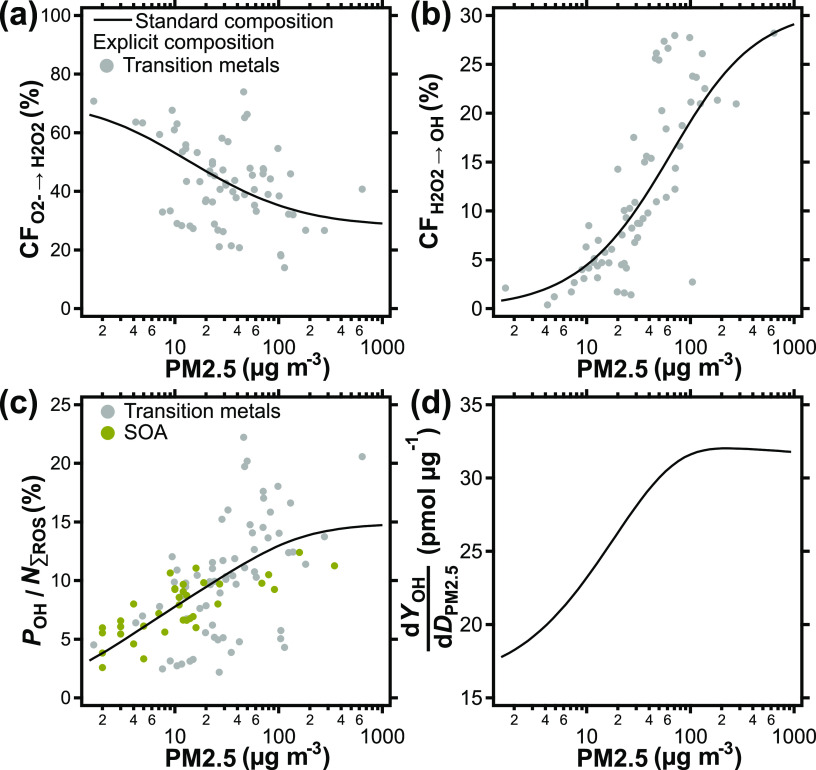
(a) ROS conversion fractions (CF) for the conversion of O_2_^–^ to H_2_O_2_ and (b) the conversion of H_2_O_2_ to OH as a function of ambient PM2.5 concentration. (c) OH fraction of the total cumulative ROS production expressed as a percentage and (d) the change in OH yield per change in PM2.5 dose in the ELF as a function of PM2.5 concentration. CF represents the fraction of the total produced ROS that undergo the indicated conversion pathway as opposed to being scavenged, exhaled, or accumulated in the ELF within 2 h of simulation time. The lines represent a standard PM2.5 composition; the markers in (a–c) show the effect of using explicit PM2.5 composition data (Table S5).

**Figure 4:**
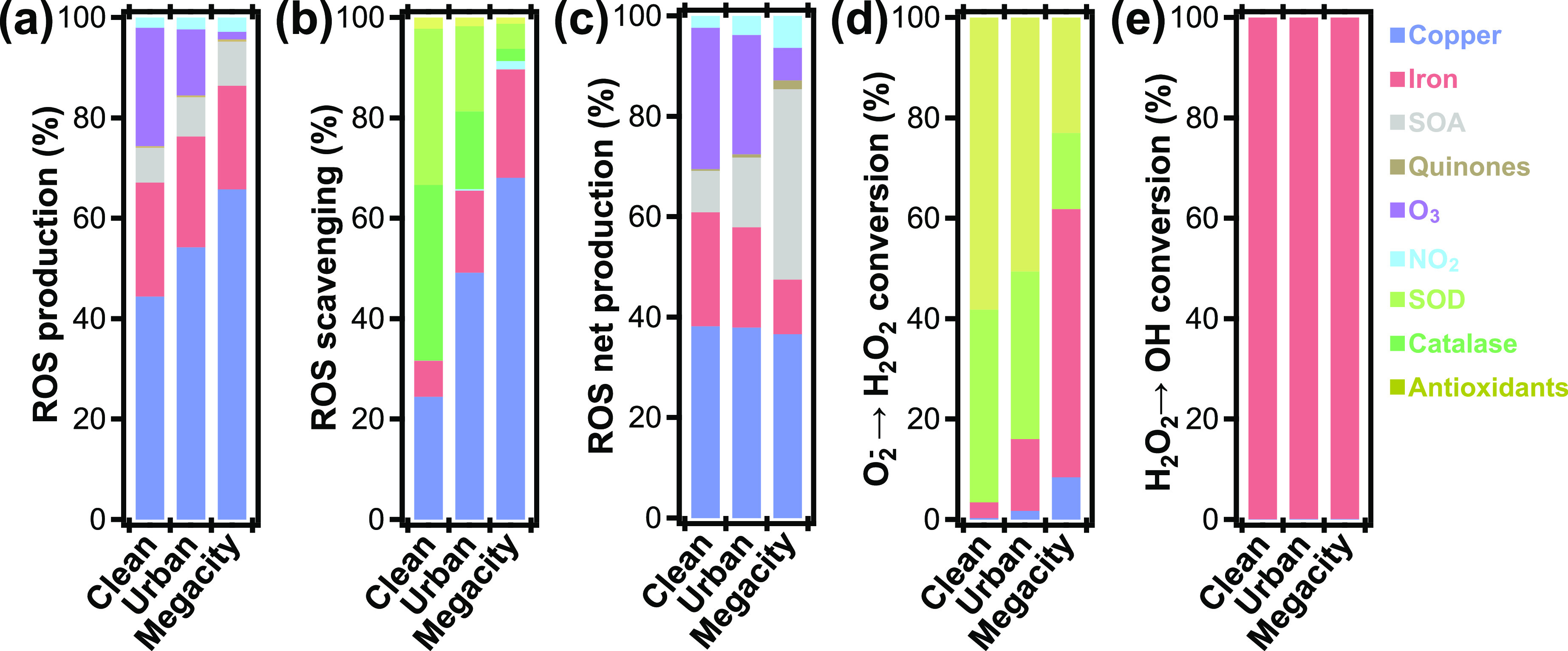
Relative contributions of pollutants, enzymes, and antioxidants to chemical production, scavenging, and conversion of ROS in ELF for three characteristic pollution scenarios (clean, urban, megacity; see the Methods section): (a) ROS production, (b) ROS scavenging, (c) ROS net production, (d) O_2_^–^-to-H_2_O_2_ conversion, and (e) H_2_O_2_-to-OH conversion. Concentrations of individual PM2.5 constituents are determined based on a standard PM2.5 composition obtained from field observations (Tables S5–S7).

To illustrate shifts in ROS interconversion patterns, a chemical pathway analysis is conducted and the results are displayed in Figure 3a,b. ROS conversion fractions (CF), i.e., the percentage fraction of a ROS that is chemically converted to other ROS and not scavenged, exhaled, or accumulated, are presented as a function of pollutant concentrations. The term scavenging is used for chemical reactions that convert ROS into largely unreactive products such as H_2_O or O_2_. Figure 3a,b shows the fraction of O_2_^–^ converted to H_2_O_2_ (CF_O2–→H2O2_) and the fraction of H_2_O_2_ converted to OH (CF_H2O2→OH_), respectively. In analogy to Figure 1, the solid lines represent standard PM2.5 composition, whereas explicit composition markers illustrate the sensitivity and variance induced by PM2.5 constituents. Explicit composition markers for SOA and quinones are omitted from Figure 3a,b as these compounds show no, or only negligible contribution to ROS interconversion, respectively. The fraction of O_2_^–^ converted to H_2_O_2_ is high with >50% at low pollution, falls below 50% at typical urban pollution levels, and stabilizes at ∼30% at very high pollution. The drop in CF_O2–__→H2O2_ can be attributed to transition metals becoming the more important reaction partner of O_2_^–^ compared to antioxidants and enzymes. The mixture of SOD and ascorbate in the ELF scavenges about 33% of O_2_^–^ and converts about 66% of O_2_^–^ to H_2_O_2_. The ratio of scavenged to converted is generally higher for transition metals, as well as depends on the iron to copper ratio, as indicated by the scatter of markers in Figure 3a. This effect will be further detailed in Figure 4d.

The terminal element in the ROS interconversion chain is the conversion of H_2_O_2_ to the OH radical. OH reacts quickly, at or near site of formation, and with nearly all molecules in the ELF, and may directly cause damage to biomolecules, cells, and tissues.^30,49^ At low PM2.5 concentrations, the fraction of H_2_O_2_ converted to OH is low, ranging from 0.5 to 4% (Figure 3b). However, this fraction shows a strong nonlinear increase from 4 to 19% at typical urban pollution levels, and may reach up to 29% at very high pollution according to the model. The nonlinear increase in CF_H2O2→OH_ with PM2.5 concentration is due to competition of catalase and transition metals for reaction with H_2_O_2_. Catalase scavenges H_2_O_2_ at a constant rate. Its importance diminishes as the rate of the Fenton reaction increases toward high PM2.5 concentrations. When PM2.5 exposure is highest, the effect of catalase is negligible and CF_H2O2→OH_ identical to the OH yield of the Fenton reaction, which is about 30% in the chemical mechanism used in this study (Table S1).

In conclusion, while the fraction of O_2_^–^ that is converted to H_2_O_2_ decreases by a factor of ∼2 over the investigated pollution range, the conversion fraction of H_2_O_2_ to OH increases by a factor of ∼50. Figure 3c shows the joint effect of ROS production and interconversion by calculating the share of OH production, *P*_OH_, within the cumulative ROS production, *N*_∑ROS_, as a function of pollutant concentration. At low pollutant concentrations, this contribution of OH to *N*_∑ROS_ is small with only ∼5%. The value increases to 15% toward very high pollution levels and may even reach 20% for specific PM2.5 compositions. This change in kinetic regime may have drastic implications on the health effects of PM2.5 as more of the highly reactive OH is created, both absolutely (Figure 2b) and relatively (Figure 3c), with increasing pollution levels. The tipping point for this regime change lies in the range of typical urban pollution in this simulation (Figure 3b).

Figure 3d shows the incremental increase in OH yield, d*Y*_OH_/d*D*_PM2.5_, plotted against PM2.5 concentration. Here, *Y*_OH_ is the OH yield (pmol) and *D*_PM2.5_ (μg) is the dose of PM2.5 inhaled and deposited in the ELF. At low pollutant concentrations, d*Y*_OH_/d*D*_PM2.5_ is around 20 pmol μg^–1^. In the range of typical urban pollutant concentrations, however, it increases steadily, suggesting that ROS buffering becomes less effective, and PM2.5 more harmful. At a pollutant concentration of 100 μg m^–3^, the incremental OH yield reaches a maximum level around 30 pmol μg^–1^. The increase of d*Y*_OH_/d*D*_PM2.5_ shows that the ROS buffering capacity of the physiological antioxidant defense is exhausted at high PM2.5 levels.

To gain insight into the chemical species and reactions responsible for this change in kinetic regime, ROS production, scavenging, and conversions are apportioned to constituents of air pollution, enzymes, and antioxidants in the ELF (Figure 4). As shown previously,^14^ copper and iron ions are found to be the main sources, i.e., gross producers of ROS in the ELF, largely independent of pollutant concentration (Figure 4a). Chemical reactions involving transition-metal ions give rise to ca. 70–90% of all initial ROS formed in the ELF by reduction of molecular oxygen O_2_ to O_2_^–^ (R48 and R54, Table S1), whereas chemical reactions involving O_3_, NO_2_, and SOA together are responsible for the remaining ca. 10–30%. O_3_ constitutes a significant ROS source in the “clean” and “urban” scenarios but is less important in the “megacity” scenario.

Figure 4b details the efficacy of all explicit ROS scavengers in the ELF. Note that, while the reaction of OH with antioxidants is counted here toward ROS scavenging, the unspecific loss of OH is not, because these reactions can retain the unpaired electron (e.g., H-abstraction: RH + OH → R• + H_2_O). These reactions may rather result in physiological damage and initiate chain propagation reactions such as lipid peroxidation.^73^ The model finds that the reactions of antioxidants with OH make up 7% of total OH loss in the ELF, which corresponds to <2% of the total ROS scavenging. The most potent endogenous ROS sinks include the enzymatic scavenging of one equivalent of O_2_^–^ in the disproportionation by superoxide dismutase (SOD, R124, Table S1), and the scavenging of H_2_O_2_ by catalase (R125, Table S1). At very low pollutant concentrations, 70% of all scavenged ROS can be attributed to reactions of enzymes, reflecting efficient ROS buffering by endogenous molecules in the ELF. However, dissolved copper and iron can also scavenge ROS (R42, 44, 51, 56, Table S1), which becomes increasingly important at higher pollutant concentrations. In the “urban” exposure scenario, already about 60% of ROS scavenging is attributed to these transition metals, whereas under “megacity” conditions this number reaches 90%. This signifies the multifaceted role of transition-metal ions in the ELF, i.e., not only inducing formation, but also loss of ROS.

To account for such multifaceted roles of pollutants, the net production of ROS from individual sources is presented in Figure 4c. Net productions are computed using the number of ROS molecules produced by a pollutant, subtracted by the number of ROS molecules scavenged in chemical reactions with that pollutant. With these considerations, the model predicts that transition-metal ions are responsible for ca. 50–60% of all ROS. While O_3_ contributes ∼30% in “clean” conditions, the contribution is reduced to less than 10% under highly polluted “megacity” conditions, where SOA becomes an important net source of ROS. Quinones are found to have a small effect on ROS formation in “megacity” conditions. Taken together, PM2.5 constituents are responsible for ∼70% of all net ROS production in “clean” conditions. This share increases to ∼80% in highly polluted “megacity” conditions.

The model simulations show that about half of the produced O_2_^–^ is scavenged during its lifetime in the ELF while the other half is converted into H_2_O_2_. Figure 4d shows that SOD (R124, Table S1) and antioxidants (R73, R74, Table S1) are the main drivers of this conversion and are responsible for over 90 and 80% of the H_2_O_2_ formation from O_2_^–^ in “clean” and “urban” environments, respectively. However, under highly polluted “megacity” conditions, transition-metal ions in PM2.5 supersede endogenous molecules in the conversion of O_2_^–^ into H_2_O_2_, which signifies another facet in the redox chemistry of transition metals in the ELF.

While multiple species in the ELF convert O_2_^–^ into H_2_O_2_, the model suggests that the conversion of H_2_O_2_ to OH almost exclusively involves the PM2.5 constituent iron (Figure 4e). This reaction converts a very stable form of ROS into a very reactive and noxious ROS, thereby strongly increasing overall ROS reactivity. Thus, the ions of the two transition metals iron and copper differ in their role for ROS formation and interconversion in the ELF: copper contributes more to initial ROS formation by reduction of O_2_ to O_2_^–^, while iron is more important for increasing ROS reactivity by conversion of H_2_O_2_ into OH radicals.^30^

**Figure 5:**
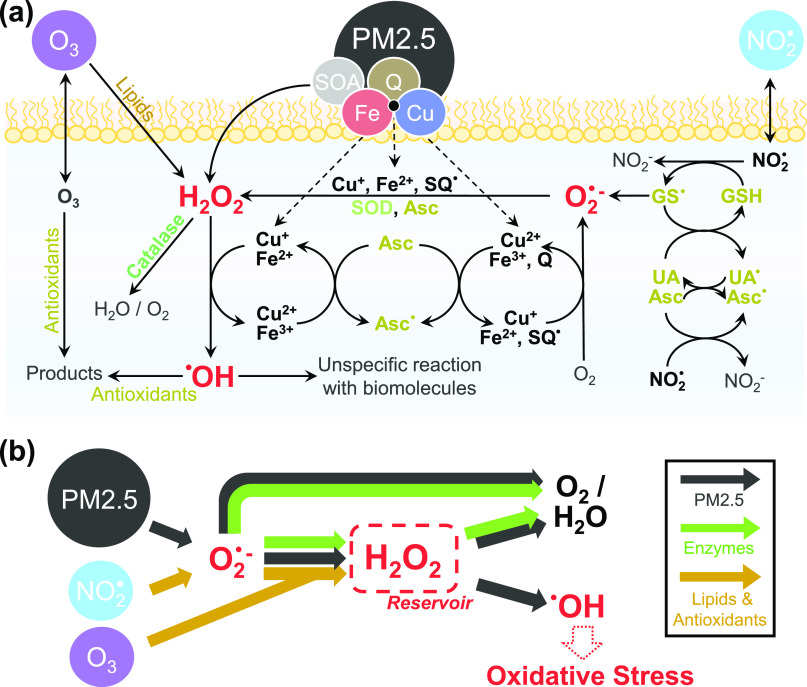
(a) Production, interconversion, and scavenging of reactive oxygen species (ROS) by air pollutants and endogenous molecules in the epithelial lining fluid (ELF). Organic and inorganic constituents of fine particulate matter (PM2.5) can produce, convert, and scavenge ROS. Enzymes (catalase; superoxide dismutase, SOD) intercept ROS through the disproportionation of O_2_^–^ and the decomposition of H_2_O_2_ (green). Antioxidants (ascorbate; glutathione, GSH; uric acid, UA; α-tocopherol, α-Toc) intercept OH, O_2_^–^, and H_2_O_2_, but the reaction of antioxidants and surfactant lipids with NO_2_ and O_3_ can also produce ROS (yellow). Note that PM2.5 constituents are able to convert the relatively stable reservoir species H_2_O_2_ into the highly reactive OH radical, which may cause oxidative stress (distress) and physiological damage.^30,79^ (b) Schematic summary of the main reaction pathways.

Figure 5a illustrates the main reaction pathways of ROS formation, interconversion, and scavenging in the ELF. Figure 5b summarizes the insights from chemical pathway analysis and apportionment as presented in Figures 1–4 in a schematic representation. In this study, we focus on OH as the main source of oxidative stress due to its unspecific, high reactivity with any biomolecule (e.g., lipids, proteins). In contrast, such reactivity is not known for other species, including O_2_^–^ and H_2_O_2_.^74^ While O_2_^–^ has been related to health effects, not many reaction rates with organic and biomolecules, other than especially redox-active substances (e.g., (semi-)quinones, thiols) and nitric oxide, are reported in the literature.^39,75−78^ Thus, O_2_^–^ may act predominantly as a transient species in the ROS interconversion chain. Similarly, H_2_O_2_ has been implicated as a mediator and marker for disease.^29,30,68,79^ While the model suggests that *C*_H2O2_ exceeds healthy levels of ∼100 nmol L^–1^ after exposure to PM2.5, H_2_O_2_ is much less reactive than other ROS, can diffuse across cells and tissues, and allows for scavenging by antioxidant enzymes.^30,80,81^ In Figure 5b, H_2_O_2_ is thus presented as a reservoir for radical species. This reservoir is pivotal in the interception of ROS by natural antioxidants and enzymes, which maintain physiological ROS concentration levels.^30,81^ ROS interception includes the conversion of O_2_^–^ into H_2_O_2_ and the scavenging of O_2_^–^ and H_2_O_2_ by antioxidants and enzymes.^30,75^ The kinetic model shows that the ELF defense mechanism against oxidative stress acts by antioxidant- and enzyme-driven conversion of the O_2_^–^ radical into H_2_O_2_, followed by enzymatic decomposition of the reservoir species to avoid conversion into the highly reactive and noxious OH radical.

In Figure 5b, the interception of ROS by natural defense mechanisms is indicated with green arrows. At PM2.5 concentrations under 10 μg m^–3^, ROS buffering is efficient and leads to low yields of OH. At PM2.5 concentrations above 10 μg m^–3^, transition-metal ions supersede SOD in its ability to intercept O_2_^–^. Furthermore, transition-metal ions compete with catalase for H_2_O_2_. When catalase is unable to remove H_2_O_2_ fast enough, substantial OH production occurs through Fenton and Fenton-like reactions. As OH cannot be effectively intercepted, Fenton chemistry circumvents ROS scavenging and reduces the ROS interception efficiency of the ELF. Thus, exposure to PM2.5 can lead to a shift from the enzyme-controlled ROS buffering regime to the PM2.5-controlled OH radical production regime, leading to increased ROS reactivity and oxidative stress. This switch in the kinetic regime to a state of diminished ROS buffering efficiency may already occur at ambient PM2.5 concentrations >10 μg m^–3^, emphasizing the need for regulators to more strictly follow the WHO air quality guideline for PM2.5 concentration, which, coincidentally, is set to 10 μg m^–3^.^82^ This is of particular importance in urban areas, in which PM2.5 concentrations often range between 10 and 100 μg m^–3^, as slight decreases in exposure levels may be especially effective in this pollution range. We note however that, to date, it remains unclear whether a safe PM2.5 pollutant concentration, at which no health effects of air pollution could be observed, exists.^1^ Although the calculated OH productions at low pollutant concentration in this study are comparatively small, they remain nonzero.

The calculations presented in this study assume a stable, physiological pH as found in healthy individuals. In certain diseased states, however, the pH of the ELF may be decreased,^83,84^ potentially exacerbating ROS formation through increased transition-metal solubility^85^ or reduced enzyme activity.^86^ Moreover, α-hydroxyhydroperoxides are suggested to increasingly produce H_2_O_2_ at low pH.^87^ To date, the exact product yields of the Fenton reaction remain unclear;^88^ however, at lower pH, the Fenton reaction may increasingly yield OH,^89,90^ which may further facilitate oxidative stress. For efficient policy-making, future studies will have to further refine the conditions, i.e., pollutant levels and composition, under which OH production in the ELF will be strongly enhanced. Factors adding uncertainty to the model are the role of ELF pH, transition-metal coordination and solubility, ELF replenishment and antioxidant recovery, OH and H_2_O_2_ yields from organic molecules and SOA, as well as concentrations of antioxidant enzymes.

In conclusion, our results suggest that the presence of PM2.5 may increasingly trigger oxidative stress in the ELF not only through an increase in overall ROS concentrations^47^ but also by increasingly producing the most noxious form of ROS, OH, in Fenton and Fenton-like reactions. Both processes, ROS production and H_2_O_2_-to-OH conversion, contribute to the exposure of biomolecules and tissues to highly reactive OH. Chemical metrics that assess the potential of air pollution to induce oxidative stress must capture both, the quantity and the overall reactivity of ROS. Hence, in this study, we introduce the metrics of cumulative ROS production (Figure 1b) and H_2_O_2_-to-OH conversion fraction (Figure 3b). It remains open how these metrics correlate with epidemiological data and disease endpoints, which will be subject to future studies.

## Supporting Information Available

ELF antioxidant concentrations (S1); ELF enzymatic reactions (S2); OH reactions with unspecified organic matter and estimated OH lifetime in the ELF (S3); particulate pollutant concentrations in the ELF (S4); gas-phase pollutant concentrations in the ELF (S5); acid dissociation (S6); pH of the ELF (S7); chemical reaction mechanism (Table S1); input parameters to the KM-SUB-ELF model (Table S2); list of symbols and definitions (Table S3); mathematical formulas used to calculate ROS metrics (Table S4); PM2.5 and transition-metal mass fractions (Table S5); PM2.5 and secondary organic aerosol (SOA) mass fractions (Table S6); PM2.5 and quinone mass fractions (Table S7); mass fractions of all redox-active PM2.5 constituents quantified in field data (Figure S1); endpoint and average ROS concentration, *C*_ΣROS_, in the ELF as a function of PM2.5 concentration (Figure S2); ROS concentration, *C*_ΣROS_, and antioxidant consumption rate as a function of pollutant concentration (Figure S3); gross chemical production of individual ROS in the ELF as a function of the concentration of three distinct pollutants (Figure S4); O_3_ and NO_2_ concentrations and saturation point in the ELF as a function of ambient pollutant concentration (Figure S5); and pH 4 sensitivity study (Figure S6) (PDF)
