# Supplementary material for: Hydroxyl Radical Production by Air Pollutants in Epithelial Lining Fluid Governed by Interconversion and Scavenging of Reactive Oxygen Species
Source: Environ Sci Technol. 2021 Oct 5;55(20):14069–79. doi: 10.1021/acs.est.1c03875 (PMC8529872; doi:10.1021/acs.est.1c03875)
Supplement: Supplementary file 1 — es1c03875_si_001.pdf [file es1c03875_si_001.pdf]

Supplementary information to

Hydroxyl radical production by air pollutants in epithelial lining fluid governed by interconversion and scavenging of reactive oxygen species

Steven Lelieveld[1], Jake Wilson[1], Eleni Dovrou[1], Ashmi Mishra[1], Pascale S. J. Lakey[2], Manabu Shiraiwa[2], Ulrich Pöschl[1], Thomas Berkemeier[1]*

[1] Multiphase Chemistry Department, Max Planck Institute for Chemistry, 55128 Mainz, Germany
[2] Department of Chemistry, University of California, Irvine, Irvine, California 92697, USA

*Correspondence to: Thomas Berkemeier (t.berkemeier@mpic.de)

Contents of Supporting Information

Additional information

S1. ELF antioxidant concentrations

S2. ELF enzymatic reactions

S3. OH reactions with unspecified organic matter and estimated OH lifetime in the ELF

S4. Particulate pollutant concentrations in the ELF

S5. Gas-phase pollutant concentrations in the ELF

S6. Acid dissociation

S7. pH of the ELF

Tables

Table S1. Chemical reactions

Table S2. Input parameters to the KM-SUB-ELF model

Table S3. List of symbols and definitions

Table S4. Mathematical formulas used to calculate ROS metrics

Table S5. PM2.5 and transition metal mass fractions

Table S6. PM2.5 and Secondary Organic Aerosol (SOA) mass fractions

Table S7. PM2.5 and quinone mass fractions

Figures

Figure S1. Mass fractions of all redox-active PM2.5 constituents quantified in field data.

Figure S2. Endpoint, and average ROS concentration, $C_{\Sigma ROS}$, in the ELF as a function of PM2.5 concentration.

Figure S3. ROS concentration, $C_{\Sigma ROS}$, and antioxidant consumption rate as a function of pollutant concentration.

Figure S4. Gross chemical production of individual ROS in the ELF as a function of the concentration of three distinct pollutants.

Figure S5. O_3 and NO_2 concentration and saturation point in the ELF as a function of ambient pollutant concentration.

Figure S6. pH 4 sensitivity study.

S1. ELF antioxidant concentrations

Four low molecular weight antioxidants are included in this study, ascorbate (AscH), glutathione (GSH), uric acid (UA) and α-tocopherol (α-Toc).[1] The concentrations of AscH, GSH, and UA in the ELF are 40 µM, 108 µM, and 200 µM, respectively.[2,3] The total α-Toc concentration in the ELF is reported as 0.7 µM,[3] however, in the presented study α-Toc is assumed to reside in the surfactant layer of the ELF with a concentration of 200 µM. It is found that pollutant exposure can lead to a spike in $C_{\Sigma ROS}$ at pollutant concentrations above 100 µg m^{-3} in the model (Fig. S3a) due to NO_2-driven depletion of ELF antioxidants within the two hours simulation time (Fig. S3b). The reaction mechanism of ROS formation by NO_2 involves formation of $GS^{\cdot}$ (R84-85, Tab. S1) and reaction to $GSSG^{\cdot -}$ (R89), leading to O_2^- production (R91).[4,5] In the presence of physiological concentrations of UA and AscH, $GS^{\cdot}$ is efficiently recycled to GSH (R96, R100) and $GSSG^{\cdot -}$ formation a minor reaction pathway. However, at low levels of UA and AscH, this O_2^- formation pathway becomes increasingly important.

Although earlier studies have shown that antioxidants can be consumed in bronchoalveolar lavage fluid after pollutant exposure,[6] a study using healthy volunteers suggested that the ELF antioxidants may not be fully depleted,[7] even after exposure to high pollutant concentrations. Kelly et al. found antioxidant concentrations to be non-zero 1.5 hours after exposure to 2 ppm NO_2 stopped, and in some cases exceeded the initial antioxidant concentration several hours after exposure due to antioxidant replenishment.[7] We are not aware of kinetic data that details the replenishment rates of antioxidants in ELF, but we can assume that they are likely fast enough to prevent full depletion of antioxidants in our two-hour simulations, preventing a spike in $C_{\Sigma ROS}$ (Fig. S3a). Thus, for simplicity, antioxidant and surfactant concentrations are assumed to stay constant in this study.

In Fig. S3b, antioxidant consumption rates are shown as a function of NO_2, PM2.5 and O_3 concentration. The dashed line represents the antioxidant consumption rate above which the initial concentration of low molecular mass antioxidants depletes within two hours of pollutant exposure. Note that, without consideration of enzymatic reactions and antioxidant replenishment, it represents a lower limit for healthy individuals.

S2. ELF enzymatic reactions

Reactions of the enzymes superoxide dismutase (SOD) and catalase are included in the chemical mechanism (R124, R125, Tab. S1). In our previous work, the SOD-catalyzed reaction of two O_2^- forming H_2O_2 and O_2 was described as a second-order reaction with respect to O_2^- and a rate coefficient of 2.70×10^{-12} cm^{-3} s^{-1}.[1,8] Dismutation of O_2^- by SOD is a multi-step process, in which one O_2^- is oxidized to O_2, another reduced to H_2O_2, and operates close to the diffusion limit.[9] Because SOD activity is often reported in terms of a single rate and turnover number, we combine both steps into one effective reaction of SOD

and O_2^- yielding 0.5 H_2O_2 and 0.5 O_2 (R124, Tab. S1). Note that this reaction is first order with respect to both, O_2^- and enzyme.

Cantin et al. (1990) showed experimentally that catalase is the most important molecule in H_2O_2 defense of the ELF, exceeding the glutathione peroxidase concentration by nearly two orders of magnitude.[10] The reaction between catalase and H_2O_2 is a two-step reaction in which a molecule of H_2O_2 reacts with catalase to form an enzyme-substrate complex, which then further reacts with another molecule of H_2O_2 to release H_2O and O_2. Because catalase activity is often reported in terms of a single rate and turnover number, we combine both steps into one effective reaction of catalase and H_2O_2, yielding 0.5 H_2O and 0.5 O_2 (R125, Tab. S1).[11]

The catalytic activity of a particular enzyme in biological samples is often reported using the enzyme unit (U) per sample volume or mass. One U is defined as the amount of enzyme needed to catalyze one micromole of substrate per minute. The catalytic activity of a biological sample is generally determined by monitoring substrate decomposition in excess of substrate. Under these conditions, catalytic activity is maximal and a first order dependence exists between the velocity of the enzymatic reaction (v_{max}, in U mL^{-1}) and the enzyme concentration (Eq. S1).[12] The proportionality factor is the turnover number k_{cat}, sometimes referred to as catalytic constant, and can be used to infer enzyme concentrations.[13]

$$[\text{Enzyme}] = \frac{v_{max}}{k_{cat}} \qquad \text{(Eq. S1)}$$

For SOD, k_{cat} is reported to range between 10^5 – 10^6 s^{-1}.[9,14] The activity of SOD in ELF was measured as 36.8 ± 2.0 *U* mL^{-1},[10] which translates to an enzyme concentration of SOD of 0.58 – 6.5 nM using Eq. S1. Note that, the enzymatic reactions in this study do not occur in excess of substrate. Thus, a second-order rate coefficient for the reaction of SOD with O_2^- of 2.65×10^{-12} cm^{-3} s^{-1} is used in the chemical mechanism (k_{R124}, Tab. S1).[15,16]

Catalase activity in ELF has been estimated to be 3.7 ± 0.6 U mL^{-1} and for k_{cat}, values in the range of 3×10^6 and 4×10^7 s^{-1} can be found.[10,13] From these numbers, we derive a concentration of catalase of 1.3 – 24 pM. Second-order rate coefficients for catalase have been measured in the range of 1.6×10^{-14} – 1.6×10^{-13} cm^{-3} s^{-1}. For this study, we use a rate coefficient of 3.2×10^{-14} cm^{-3} s^{-1} (k_{R125}, Tab. S1).[11,17]

Given the broad reported ranges of v_{max} and k_{cat}, and the difficulty of sampling ELF, we acknowledge that there is large uncertainty associated with the concentrations of SOD and catalase in the ELF. Therefore, we restrict the determination of enzyme concentration to order of magnitude estimations and use 1 nM for SOD and 5 pM for catalase in this study.

Note that the presence of PM may decrease the activity of antioxidant enzymes, as shown for SOD previously.[18] At the high PM concentrations for which these effects were observed, however, antioxidant

enzymes contribute only marginally to overall ROS scavenging and interconversion in the model (Figs. 4b,d). Thus, enzyme inhibition by PM2.5 requires further investigation in future experimental and modelling studies to clarify whether enzyme inhibition promotes the shift to the PM2.5-dominated kinetic regime of ROS conversion.

S3. Unspecific reaction of OH radicals with organic matter and estimated OH lifetime in the ELF

OH reacts with nearly all matter present in the ELF with a rate coefficient approaching diffusion limitation.[19] Because of this unspecific reactivity, effective scavenging of OH radicals, e.g. through lung antioxidants, is not possible.[19,20] As an estimate, we assume that the amount of protein in the ELF corresponds to the total amount of dissolved organic matter. The protein mass in the ELF amounts to approximately 10 mg per mL lung fluid.[3] Using an average molecular weight of ~125 g $mole^{-1}$ of a single amino acid, the total amino acid concentration in the ELF can be estimated to ~80 mmol L^{-1}. A second-order reaction of amino acids with OH is included, using a reaction rate coefficient on the order of 1.66×10^{-12} cm^{-3} s^{-1} (R122, Tab. S1).[21] Because proteins are folded, not all amino acids will be surface exposed, and thus accessible reaction partners for OH. In general, spherical proteins have fewer surface exposed amino acids due to a smaller surface-to-volume ratio, while elongated, cuboid or conical proteins have more surface exposed amino acids. Furthermore, the surface exposure of amino acids depends on the physical properties, e.g. the polarity of the respective amino acid.[22] Therefore, as an order of magnitude estimation, we assume that 50% of all amino acids are surface exposed in the ELF, yielding an effective amino acid concentration of ~40 mmol L^{-1} and, in turn, a lifetime of OH with respect to reaction with dissolved organic matter of 2.5×10^{-8} s.

Pryor estimated the lifetime of OH in a cell to 10^{-9} s, assuming a rate coefficient of 1×10^{9} M^{-1} s^{-1} (equivalent to 1.66×10^{-12} cm^{-3} s^{-1}) and an effective organic matter concentration of 1 mol L^{-1}.[21] From comparing the ELF protein mass of ~10 mg mL^{-1} to the cellular protein mass of ~250 mg mL^{-1}, we infer that the ELF must be about ~25 times more dilute compared to a cell with respect to dissolved organic matter.[2,23,24] Multiplying Pryor's OH lifetime in cells with this dilution factor yields an estimate for the OH lifetime in ELF of ~2.5×10^{-8} s, which is identical to the estimate above and consolidates our description of OH reactivity.

We find that inclusion of this second-order loss reaction of OH results in a decrease of momentary OH concentrations by one order of magnitude compared to our earlier calculations (Fig. 2a).[1] This finding suggests that OH will react unspecifically with organic matter and only secondarily with antioxidants (7%) in the ELF. Due to the fast reaction of OH, spatial gradients of reactants could play a role in OH fate, e.g. through local depletion of antioxidants around a dissolving particle or inhomogeneous distribution of organic matter and PM2.5 constituents in the ELF. However, for the calculations in this study, starting concentrations of antioxidants and organic matter were homogeneous across the bulk ELF.

S4. Particulate pollutant concentrations in the ELF

The ELF concentrations of redox-active PM2.5 constituents, $C_{\mathrm{ELF,Y}}$, with Y standing for copper, iron, SOA, or quinones, are calculated using Eq. S2.[1]

$$C_Y = \frac{C_{gas,PM2.5} \times VR \times t_{acc} \times d_{PM2.5} \times MF_Y \times SF_Y}{M_Y \times V_{ELF}} \quad \text{(Eq. S2)}$$

Inhaled particles can reside in the ELF for several hours.[25] For this study, we assume a residence time of PM2.5 of 2 hours and use this as accumulation time of inhaled particles (t_{acc}) and simulation time (t_{sim}) to mimic a pseudo steady-state of ROS concentrations that would be achieved through continuous inhalation, in line with our previous studies.[1] It should be noted that there is some uncertainty regarding the residence time of PM2.5 in the ELF, with estimates on PM2.5 clearance also exceeding 2 hours.[26] As lung ventilation rate, VR, 1.5 $m^3\ h^{-1}$ is used, and the PM2.5 deposition fraction in the ELF, $d_{\mathrm{PM2.5}}$, is assumed to be 0.45.[1,27] The total ELF volume, V_{ELF}, is set to 20 mL.[1,28] This study only includes copper and iron as transition metals, because these are the only two that have been shown to significantly produce ROS in surrogate ELF (sELF).[29,30] The fractional solubilities, SF_{Y}, of copper and iron ions were discussed and tested extensively in our previous study, including the effects of varying trace metal solubilities.[1] In line with the main results of our previous study, the fractional solubilities of copper and iron ions are set to 0.4, 0.1, respectively.[1] Due to the relatively small concentration of iron ions and the large concentration of potential organic ligands, iron ions are expected not to precipitate in the ELF.[31] For the organic fraction in PM2.5, full solubility is assumed. Mass fractions (*MF*) of redox-active PM2.5 constituents are derived from field observations and tabulated in Tables S4-S6. Not all references in Tables S4 and S6 include PM2.5 concentrations. In such cases, PM2.5 concentrations are estimated based on similar geographical locations and indicated with an asterisk. Additionally, secondary organic aerosol (SOA) forms ROS and is included in the model.[32–34] However, because the exact mechanism of ROS formation by SOA in the ELF, first order formation rates of H_2O_2 and OH by SOA were parameterized based on experimental observations.[1,32,33] Quinones in PM2.5 are included in this study as previously described.[1] Three quinones are included that were shown to form ROS in sELF, phenanthrenequinone (PQN), 1,2-naphthoquinone (1,2-NQN), and 1,4-naphthoquinone (1,4-NQN) in a molar ratio of 2:1:1.[29]

S5. Gas-phase pollutant concentrations in the ELF

Exposure to gas-phase oxidants, O_3 and NO_2 is quantified in the model using a simplified breathing mechanism. In our previous study, it was assumed that the concentrations of these gas-phase oxidants in the lung were equal to their respective ambient concentrations. However, because of the reactivity of these oxidants, we find that lung gas-phase concentrations of these oxidants are depressed and limited by supply from inhalation of ambient air. In order to get accurate estimates for the amount of gas-phase oxidants

transferred to the surfactant layer and ELF, an average lung volume of four L, an average breath volume of 1.5 L and an average duration of a breath of 3.6 s are used to compute mass fluxes into and out of the lung (Table S2). Application of this simplified breathing mechanism results in a significant decrease in the amount of gaseous oxidants in the surfactant layer and ELF. Therefore, neither O_3, nor NO_2 are saturated in ELF with respect to their ambient concentrations in this study (Fig. S5).

S6. Acid dissociation

In this study, corresponding acid/base-pairs are treated as a single species in the numerical computation of ordinary differential equations (ODE). This effectively reduces the stiffness of the ODE system and applies to glutathione (GSH/GS^-), superoxide radicals (HO_2/O_2^-) and peroxynitrous acid ($ONOOH/ONOO^-$). Instead of treating each species explicitly with separate differential equations and explicit protonation and deprotonation reactions, the pKa of these species was used to calculate the acid/base-ratio at the pH of the ELF (Table S2). Then, if a reaction requires only one of the two species to react, the rate of that reaction was multiplied with the inferred fraction of the reacting species.

S7. pH of the ELF

Following estimations by Holma (1985, 1989), the pH of the ELF was assumed to stay constant upon air pollutant exposure.[35,36] In diseased individuals such as asthmatics[37], chronic obstructive pulmonary disease,[38] or cystic fibrosis patients[39] the pH of the ELF may be decreased. Figure S6 shows the ROS concentration, production, interconversion and transition metal valence state at pH 7 and pH 4 as a function of PM2.5 concentration. Panel a shows that $C_{\Sigma ROS}$ displays a very similar behavior at pH 4 and pH 7. C_{O2-} and C_{HO2} are slightly increased at low PM2.5 concentrations, but depressed at elevated PM2.5 concentrations (panel b). This reduction is due to a higher rate of Fe^{2+}-mediated interconversion of HO_2 and O_2^- to H_2O_2 (Table S1, R38 and R39), which in turn is due to a higher Fe^{2+}/Fe^{3+} ratio (panel c). The Cu^+/Cu^{2+} ratio shows the opposite trend at reduced pH (panel j).

Panel e shows that at pH 4, $N_{\Sigma ROS}$ is marginally reduced compared to pH 7. In panel f, $N_{\Sigma ROS}$ is broken down to its components. P_{O2-} is slightly reduced due to decreased Cu^+-dependent O_2^- formation (Table S1, R54). P_{OH} is slightly increased due to the higher Fe^{2+}/Fe^{3+} ratio and the according increase in the Fenton reaction (Table S1, R40).

Panel g shows that $CF_{O2- \rightarrow H2O2}$ is slightly decreased at low PM2.5 concentration and slightly increased at higher PM2.5 concentrations compared to pH 7. Panel h shows that $CF_{H2O2 \rightarrow OH}$ is mostly unaffected by a change in pH. The reduction in $N_{\Sigma ROS}$ (panel e) paired with the increase in P_{OH} (panel f) at higher PM2.5 concentrations results in a larger OH fraction (panel i) and a larger increase in OH dose (panel j) at pH 4.

Reduced pH in the ELF may additionally lead to a reduction in antioxidant enzyme activity,[17] increased transition metal solubility,[40] and increased OH yield from the Fenton reaction.[41,42] These effects are not included in the presented study, and are expected to all reduce ROS buffering and promote the PM2.5-

controlled OH radical production regime, which may exacerbate oxidative stress. Furthermore, in the presented study SOA produces H_2O_2 and OH,[32,33] which at lower pH may increasingly shift towards only H_2O_2 production, without OH getting formed.[43,44] However, a thorough investigation of pH effects is beyond the scope of the presented study and warrants future investigations.

References

(1) Lakey, P. S. J.; Berkemeier, T.; Tong, H.; Arangio, A. M.; Lucas, K.; Pöschl, U.; Shiraiwa, M. Chemical Exposure-Response Relationship between Air Pollutants and Reactive Oxygen Species in the Human Respiratory Tract. *Sci. Rep.* **2016**, *6* (1), 32916. https://doi.org/10.1038/srep32916.

(2) Mudway, I. S.; Kelly, F. J. Ozone and the Lung: A Sensitive Issue. *Mol. Aspects Med.* **2000**, *21* (1–2), 1–48. https://doi.org/10.1016/S0098-2997(00)00003-0.

(3) van der Vliet, A.; O'Neill, C. A.; Cross, C. E.; Koostra, J. M.; Volz, W. G.; Halliwell, B.; Louie, S. Determination of Low-Molecular-Mass Antioxidant Concentrations in Human Respiratory Tract Lining Fluids. *Am. J. Physiol.-Lung Cell. Mol. Physiol.* **1999**, *276* (2), L289–L296. https://doi.org/10.1152/ajplung.1999.276.2.L289.

(4) Kirsch, M.; Lehnig, M.; Korth, H.-G.; Sustmann, R.; de Groot, H. Inhibition of Peroxynitrite-Induced Nitration of Tyrosine by Glutathione in the Presence of Carbon Dioxide through Both Radical Repair and Peroxynitrate Formation. *Chem. Eur. J.* **2001**, *7* (15), 3313–3320. https://doi.org/10.1002/1521-3765(20010803)7:15<3313::aid-chem3313>3.0.co;2-7.

(5) Wardman, P.; Sonntag, C. [3] Kinetic factors that control the fate of thiyl radicals in cells. In *Methods in Enzymology*; Elsevier: gr, 1995; Vol. 251, pp 31–45. https://doi.org/10.1016/0076-6879(95)51108-3.

(6) Kelly, F. J.; Tetley, T. D. Nitrogen Dioxide Depletes Uric Acid and Ascorbic Acid but Not Glutathione from Lung Lining Fluid. *Biochem. J.* **1997**, *325* (1), 95–99. https://doi.org/10.1042/bj3250095.

(7) Kelly, F. J.; Blomberg, A.; Frew, A.; Holgate, S. T.; Sandstrom, T. Antioxidant Kinetics in Lung Lavage Fluid Following Exposure of Humans to Nitrogen Dioxide. *Am. J. Respir. Crit. Care Med.* **1996**, *154* (6), 1700–1705. https://doi.org/10.1164/ajrccm.154.6.8970358.

(8) Kohen, R.; Nyska, A. Invited Review: Oxidation of Biological Systems: Oxidative Stress Phenomena, Antioxidants, Redox Reactions, and Methods for Their Quantification. *Toxicol. Pathol.* **2002**, *30* (6), 620–650. https://doi.org/10.1080/01926230290166724.

(9) Fee, J. A.; Bull, C. Steady-State Kinetic Studies of Superoxide Dismutases. Saturative Behavior of the Copper- and Zinc-Containing Protein. *J. Biol. Chem.* **1986**, *261* (28), 13000–13005. https://doi.org/10.1016/S0021-9258(18)69261-0.

(10) Cantin, A. M.; Fells, G. A.; Hubbard, R. C.; Crystal, R. G. Antioxidant Macromolecules in the Epithelial Lining Fluid of the Normal Human Lower Respiratory Tract. *J. Clin. Invest.* **1990**, *86* (3), 962–971. https://doi.org/10.1172/JCI114798.

(11) Aebi, H. [13] Catalase in Vitro. *Methods Enzymol.* **1984**, *105*, 121–126.

(12) Bisswanger, H. *Practical Enzymology*; John Wiley & Sons, 2019.

(13) Smejkal, G. B.; Kakumanu, S. Enzymes and Their Turnover Numbers. *Expert Rev. Proteomics* **2019**, *16* (7), 543–544. https://doi.org/10.1080/14789450.2019.1630275.

(14) Bar-Even, A.; Noor, E.; Savir, Y.; Liebermeister, W.; Davidi, D.; Tawfik, D. S.; Milo, R. The Moderately Efficient Enzyme: Evolutionary and Physicochemical Trends Shaping Enzyme Parameters. *Biochemistry* **2011**, *50* (21), 4402–4410. https://doi.org/10.1021/bi2002289.

(15) Forman, H. J.; Fridovich, I. Superoxide Dismutase: A Comparison of Rate Constants. *Arch. Biochem. Biophys.* **1973**, *158* (1), 396–400. https://doi.org/10.1016/0003-9861(73)90636-X.

(16) Fridovich, I. Superoxide Dismutases. *Annu. Rev. Biochem.* **1975**, *44* (1), 147–159.

(17) Jones, P.; Suggett, A. The Catalase–Hydrogen Peroxide System. Kinetics of Catalatic Action at High Substrate Concentrations. *Biochem. J.* **1968**, *110* (4), 617–620. https://doi.org/10.1042/bj1100617.

(18) Hatzis, C.; Godleski, J. J.; González-Flecha, B.; Wolfson, J. M.; Koutrakis, P. Ambient Particulate Matter Exhibits Direct Inhibitory Effects on Oxidative Stress Enzymes. *Environ. Sci. Technol.* **2006**, *40* (8), 2805–2811. https://doi.org/10.1021/es0518732.

(19) Forman, H. J.; Davies, K. J. A.; Ursini, F. How Do Nutritional Antioxidants Really Work: Nucleophilic Tone and Para-Hormesis versus Free Radical Scavenging in Vivo. *Free Radic. Biol. Med.* **2014**, *66*, 24–35. https://doi.org/10.1016/j.freeradbiomed.2013.05.045.

(20) Sies, H.; Berndt, C.; Jones, D. P. Oxidative Stress. *Annu. Rev. Biochem.* **2017**, *86*, 715–748. https://doi.org/10.1146/annurev-biochem-061516-045037.

(21) Pryor, W. A. Oxy-Radicals and Related Species: Their Formation, Lifetimes, and Reactions. *Annu. Rev. Physiol.* **1986**, *48* (1), 657–667.

(22) Holbrook, S. R.; Muskal, S. M.; Kim, S.-H. Predicting Surface Exposure of Amino Acids from Protein Sequence. *Protein Eng. Des. Sel.* **1990**, *3* (8), 659–665. https://doi.org/10.1093/protein/3.8.659.

(23) Milo, R. What Is the Total Number of Protein Molecules per Cell Volume? A Call to Rethink Some Published Values. *BioEssays* **2013**, *35* (12), 1050–1055. https://doi.org/10.1002/bies.201300066.

(24) Brown, G. C. Total Cell Protein Concentration as an Evolutionary Constraint on the Metabolic Control Distribution in Cells. *J. Theor. Biol.* **1991**, *153* (2), 195–203. https://doi.org/10.1016/S0022-5193(05)80422-9.

(25) Ghio, A. J.; Richards, J. H.; Dittrich, K. L.; Samet, J. M. Metal Storage and Transport Proteins Increase After Exposure of the Rat Lung to an Air Pollution Particle. *Toxicol. Pathol.* **1998**, *26* (3), 388–394. https://doi.org/10.1177/019262339802600313.

(26) Lippmann, M.; Yeates, D. B.; Albert, R. E. Deposition, Retention, and Clearance of Inhaled Particles. *Occup. Environ. Med.* **1980**, *37* (4), 337–362. https://doi.org/10.1136/oem.37.4.337.

(27) Sarangapani, R. The Role of Dispersion in Particle Deposition in Human Airways. *Toxicol. Sci.* **2000**, *54* (1), 229–236. https://doi.org/10.1093/toxsci/54.1.229.

(28) Walters, D. V. Lung Lining Liquid – The Hidden Depths. *Neonatology* **2002**, *81* (1), 2–5. https://doi.org/10.1159/000056764.

(29) Charrier, J. G.; McFall, A. S.; Richards-Henderson, N. K.; Anastasio, C. Hydrogen Peroxide Formation in a Surrogate Lung Fluid by Transition Metals and Quinones Present in Particulate Matter. *Environ. Sci. Technol.* **2014**, *48* (12), 7010–7017. https://doi.org/10.1021/es501011w.

(30) Charrier, J. G.; Anastasio, C. Impacts of Antioxidants on Hydroxyl Radical Production from Individual and Mixed Transition Metals in a Surrogate Lung Fluid. *Atmos. Environ.* **2011**, *45* (40), 7555–7562. https://doi.org/10.1016/j.atmosenv.2010.12.021.

(31) Gonzalez, D. H.; Diaz, D. A.; Baumann, J. P.; Ghio, A. J.; Paulson, S. E. Effects of Albumin, Transferrin and Humic-like Substances on Iron-Mediated OH Radical Formation in Human Lung Fluids. *Free Radic. Biol. Med.* **2021**, *165*, 79–87. https://doi.org/10.1016/j.freeradbiomed.2021.01.021.

(32) Wang, Y.; Kim, H.; Paulson, S. E. Hydrogen Peroxide Generation from α- and β-Pinene and Toluene Secondary Organic Aerosols. *Atmos. Environ.* **2011**, *45* (18), 3149–3156. https://doi.org/10.1016/j.atmosenv.2011.02.060.

(33) Tong, H.; Arangio, A. M.; Lakey, P. S. J.; Berkemeier, T.; Liu, F.; Kampf, C. J.; Brune, W. H.; Pöschl, U.; Shiraiwa, M. Hydroxyl Radicals from Secondary Organic Aerosol Decomposition in Water. *Atmospheric Chem. Phys.* **2016**, *16* (3), 1761–1771. https://doi.org/10.5194/acp-16-1761-2016.

(34) Tong, H.; Lakey, P. S. J.; Arangio, A. M.; Socorro, J.; Kampf, C. J.; Berkemeier, T.; Brune, W. H.; Pöschl, U.; Shiraiwa, M. Reactive Oxygen Species Formed in Aqueous Mixtures of Secondary Organic Aerosols and Mineral Dust Influencing Cloud Chemistry and Public Health in the Anthropocene. *Faraday Discuss.* **2017**, *200*, 251–270. https://doi.org/10.1039/C7FD00023E.

(35) Holma, B. Influence of Buffer Capacity and PH-Dependent Rheological Properties of Respiratory Mucus on Health Effects Due to Acidic Pollution. *Sci. Total Environ.* **1985**, *41* (2), 101–123. https://doi.org/10.1016/0048-9697(85)90181-0.
(36) Holma, B. Effects of Inhaled Acids on Airway Mucus and Its Consequences for Health. *Env. Health Perspect* **1989**, No. 79, 109–113.
(37) Hunt, J. F.; Fang, K.; Malik, R.; Snyder, A.; Malhotra, N.; Platts-Mills, T. A. E.; Gaston, B. Endogenous Airway Acidification: Implications for Asthma Pathophysiology. *Am. J. Respir. Crit. Care Med.* **2000**, *161* (3), 694–699. https://doi.org/10.1164/ajrccm.161.3.9911005.
(38) Ricciardolo, F. L. M.; Gaston, B.; Hunt, J. Acid Stress in the Pathology of Asthma. *J. Allergy Clin. Immunol.* **2004**, *113* (4), 610–619. https://doi.org/10.1016/j.jaci.2003.12.034.
(39) Tate, S.; MacGregor, G.; Davis, M.; Innes, J.; Greening, A. Airways in Cystic Fibrosis Are Acidified: Detection by Exhaled Breath Condensate. *Thorax* **2002**, *57* (11), 926–929.
(40) Fang, T.; Guo, H.; Zeng, L.; Verma, V.; Nenes, A.; Weber, R. J. Highly Acidic Ambient Particles, Soluble Metals, and Oxidative Potential: A Link between Sulfate and Aerosol Toxicity. *Environ. Sci. Technol.* **2017**, *51* (5), 2611–2620. https://doi.org/10.1021/acs.est.6b06151.
(41) Hug, S. J.; Leupin, O. Iron-Catalyzed Oxidation of Arsenic(III) by Oxygen and by Hydrogen Peroxide: PH-Dependent Formation of Oxidants in the Fenton Reaction. *Environ. Sci. Technol.* **2003**, *37* (12), 2734–2742. https://doi.org/10.1021/es026208x.
(42) Bataineh, H.; Pestovsky, O.; Bakac, A. PH-Induced Mechanistic Changeover from Hydroxyl Radicals to Iron(Iv) in the Fenton Reaction. *Chem. Sci.* **2012**, *3* (5), 1594. https://doi.org/10.1039/c2sc20099f.
(43) Qiu, J.; Tonokura, K.; Enami, S. Proton-Catalyzed Decomposition of α-Hydroxyalkyl-Hydroperoxides in Water. *Environ. Sci. Technol.* **2020**, *54* (17), 10561–10569. https://doi.org/10.1021/acs.est.0c03438.
(44) Enami, S. Fates of Organic Hydroperoxides in Atmospheric Condensed Phases. *J. Phys. Chem. A* **2021**, *125* (21), 4513–4523. https://doi.org/10.1021/acs.jpca.1c01513.
(45) Saunders, S. M.; Jenkin, M. E.; Derwent, R. G.; Pilling, M. J. Protocol for the Development of the Master Chemical Mechanism, MCM v3 (Part A): Tropospheric Degradation of Non-Aromatic Volatile Organic Compounds. *Atmos Chem Phys* **2003**, *3* (1), 161–180. https://doi.org/10.5194/acp-3-161-2003.
(46) Jenkin, M. E.; Saunders, S. M.; Wagner, V.; Pilling, M. J. Protocol for the Development of the Master Chemical Mechanism, MCM v3 (Part B): Tropospheric Degradation of Aromatic Volatile Organic Compounds. *Part B* **2003**, *3* (1), 181–193. https://doi.org/10.5194/acp-3-181-2003.
(47) Buxton, G. V.; Greenstock, C. L.; Helman, W. P.; Ross, A. B. Critical Review of Rate Constants for Reactions of Hydrated Electrons, Hydrogen Atoms and Hydroxyl Radicals (·OH/·O^- in Aqueous Solution. *J. Phys. Chem. Ref. Data* **1988**, *17* (2), 513–886. https://doi.org/10.1063/1.555805.
(48) Hoffman, M. Z.; Hayon, E. Pulse Radiolysis Study of Sulfhydryl Compounds in Aqueous Solution. *J. Phys. Chem.* **1973**, *77* (8), 990–996. https://doi.org/10.1021/j100627a005.
(49) Zhao, M. J.; Jung, L.; Tanielian, C.; Mechin, R. Kinetics of the Competitive Degradation of Deoxyribose and Other Biomolecules by Hydroxyl Radicals Produced by the Fenton Reaction. *Free Radic. Res.* **1994**, *20* (6), 345–363. https://doi.org/10.3109/10715769409145635.
(50) Kanofsky, J. R.; Sima, P. D. Reactive Absorption of Ozone by Aqueous Biomolecule Solutions: Implications for the Role of Sulfhydryl Compounds as Targets for Ozone. *Arch. Biochem. Biophys.* **1995**, *316* (1), 52–62. https://doi.org/10.1006/abbi.1995.1009.
(51) Pryor, W. A.; Giamalva, D. H.; Church, D. F. Kinetics of Ozonation. 2. Amino Acids and Model Compounds in Water and Comparisons to Rates in Nonpolar Solvents. *J. Am. Chem. Soc.* **1984**, *106* (23), 7094–7100. https://doi.org/10.1021/ja00335a038.

(52) Kim, H. I.; Kim, H.; Shin, Y. S.; Beegle, L. W.; Jang, S. S.; Neidholdt, E. L.; Goddard, W. A.; Heath, J. R.; Kanik, I.; Beauchamp, J. L. Interfacial Reactions of Ozone with Surfactant Protein B in a Model Lung Surfactant System. *J. Am. Chem. Soc.* **2010**, *132* (7), 2254–2263. https://doi.org/10.1021/ja908477w.

(53) Hasson, A. S.; Ho, A. W.; Kuwata, K. T.; Paulson, S. E. Production of Stabilized Criegee Intermediates and Peroxides in the Gas Phase Ozonolysis of Alkenes: 2. Asymmetric and Biogenic Alkenes. *J. Geophys. Res. Atmospheres* **2001**, *106* (D24), 34143–34153. https://doi.org/10.1029/2001JD000598.

(54) Hewitt, C. N.; Kok, G. L. Formation and Occurrence of Organic Hydroperoxides in the Troposphere: Laboratory and Field Observations. *J. Atmospheric Chem.* **1991**, *12* (2), 181–194. https://doi.org/10.1007/BF00115779.

(55) Zhou, Z.; Abbatt, J. P. D. Formation of Gas-Phase Hydrogen Peroxide via Multiphase Ozonolysis of Unsaturated Lipids. *Environ. Sci. Technol. Lett.* **2021**, *8* (2), 114–120. https://doi.org/10.1021/acs.estlett.0c00757.

(56) Navarrete, M.; Rangel, C.; Corchado, J. C.; Espinosa-García, J. Trapping of the OH Radical by α-Tocopherol: A Theoretical Study. *J. Phys. Chem. A* **2005**, *109* (21), 4777–4784. https://doi.org/10.1021/jp050717e.

(57) Kermani, S.; Ben-Jebria, A.; Ultman, J. S. Kinetics of Ozone Reaction with Uric Acid, Ascorbic Acid, and Glutathione at Physiologically Relevant Conditions. *Arch. Biochem. Biophys.* **2006**, *451* (1), 8–16. https://doi.org/10.1016/j.abb.2006.04.015.

(58) Rush, J. D.; Bielski, B. H. J. Pulse Radiolytic Studies of the Reaction of Perhydroxyl/Superoxide O2- with Iron(II)/Iron(III) Ions. The Reactivity of HO2/O2- with Ferric Ions and Its Implication on the Occurrence of the Haber-Weiss Reaction. *J. Phys. Chem.* **1985**, *89* (23), 5062–5066. https://doi.org/10.1021/j100269a035.

(59) Christensen, H.; Sehested, K.; Corfitzen, H. Reactions of Hydroxyl Radicals with Hydrogen Peroxide at Ambient and Elevated Temperatures. *J. Phys. Chem.* **1982**, *86* (9), 1588–1590. https://doi.org/10.1021/j100206a023.

(60) Sehested, K.; Rasmussen, O. L.; Fricke, H. Rate Constants of OH with HO2,O2-, and H2O2+ from Hydrogen Peroxide Formation in Pulse-Irradiated Oxygenated Water. *J. Phys. Chem.* **1968**, *72* (2), 626–631. https://doi.org/10.1021/j100848a040.

(61) Koppenol, W. H. The Haber-Weiss Cycle – 70 Years Later. *Redox Rep.* **2001**, *6* (4), 229–234. https://doi.org/10.1179/135100001101536373.

(62) Jayson, G. G.; Parsons, B. J.; Swallow, A. J. Oxidation of Ferrous Ions by Perhydroxyl Radicals. *J. Chem. Soc. Faraday Trans. 1 Phys. Chem. Condens. Phases* **1973**, *69*, 236–242. https://doi.org/10.1039/f19736900236.

(63) Lewis, S.; Lynch, A.; Bachas, L.; Hampson, S.; Ormsbee, L.; Bhattacharyya, D. Chelate-Modified Fenton Reaction for the Degradation of Trichloroethylene in Aqueous and Two-Phase Systems. *Environ. Eng. Sci.* **2009**, *26* (4), 849–859. https://doi.org/10.1089/ees.2008.0277.

(64) Stuglik, Z.; PawełZagórski, Z. Pulse Radiolysis of Neutral Iron(II) Solutions: Oxidation of Ferrous Ions by OH Radicals. *Radiat. Phys. Chem. 1977* **1981**, *17* (4), 229–233. https://doi.org/10.1016/0146-5724(81)90336-8.

(65) Masuda, T.; Shinohara, H.; Kondo, M. Reactions of Hydroxyl Radicals with Nucleic Acid Bases and the Related Compounds in Gamma-Irradiated Aqueous Solution. *J. Radiat. Res. (Tokyo)* **1978**, *16* (3), 153–161.

(66) Liphard, M.; Bothe, E.; Schulte-Frohlinde, D. The Influence of Glutathione on Single-Strand Breakage in Single-Stranded DNA Irradiated in Aqueous Solution in the Absence and Presence of Oxygen. *Int. J. Radiat. Biol.* **1990**, *58* (4), 589–602. https://doi.org/10.1080/09553009014551951.

(67) Carr, A.; Lykkesfeldt, J. *Vitamin C in Health and Disease*; MDPI-Multidisciplinary Digital Publishing Institute, 2018.

(68) Shen, J.; Griffiths, P. T.; Campbell, S. J.; Utinger, B.; Kalberer, M.; Paulson, S. E. Ascorbate Oxidation by Iron, Copper and Reactive Oxygen Species: Review, Model Development, and Derivation of Key Rate Constants. *Sci. Rep.* **2021**, *11* (1), 7417. https://doi.org/10.1038/s41598-021-86477-8.

(69) Adams, G. E.; Boag, J. W.; Currant, J.; Michael, B. D. *Absolute Rate Constants for the Reaction of the Hydroxyl Radical with Organic Compounds*; Pulse Radiolysis, 1965.

(70) Goldstein, S.; Lind, J.; Merenyi, G. Reaction of Organic Peroxyl Radicals with $^{\bullet}NO_2$ and $^{\bullet}NO$ in Aqueous Solution: Intermediacy of Organic Peroxynitrate and Peroxynitrite Species. *J. Phys. Chem. A* **2004**, *108* (10), 1719–1725. https://doi.org/10.1021/jp037431z.

(71) Jones, C. M.; Lawrence, A.; Wardman, P.; Burkitt, M. J. Electron Paramagnetic Resonance Spin Trapping Investigation into the Kinetics of Glutathione Oxidation by the Superoxide Radical: Re-Evaluation of the Rate Constant. *Free Radic. Biol. Med.* **2002**, *32* (10), 982–990. https://doi.org/10.1016/S0891-5849(02)00791-8.

(72) Winterbourn, C. C.; Metodiewa, D. The Reaction of Superoxide with Reduced Glutathione. *Arch. Biochem. Biophys.* **1994**, *314* (2), 284–290. https://doi.org/10.1006/abbi.1994.1444.

(73) Wefers, H.; Sies, H. Oxidation of Glutathione by the Superoxide Radical to the Disulfide and the Sulfonate Yielding Singlet Oxygen. *Eur. J. Biochem.* **1983**, *137* (1–2), 29–36. https://doi.org/10.1111/j.1432-1033.1983.tb07791.x.

(74) Ford, E.; Hughes, M. N.; Wardman, P. Kinetics of the Reactions of Nitrogen Dioxide with Glutathione, Cysteine, and Uric Acid at Physiological PH. *Free Radic. Biol. Med.* **2002**, *32* (12), 1314–1323. https://doi.org/10.1016/S0891-5849(02)00850-X.

(75) Luo, D.; Smith, S. W.; Anderson, B. D. Kinetics and Mechanism of the Reaction of Cysteine and Hydrogen Peroxide in Aqueous Solution. *J. Pharm. Sci.* **2005**, *94* (2), 304–316. https://doi.org/10.1002/jps.20253.

(76) Winkler, B. S.; Orselli, S. M.; Rex, T. S. The Redox Couple between Glutathione and Ascorbic Acid: A Chemical and Physiological Perspective. *Free Radic. Biol. Med.* **1994**, *17* (4), 333–349. https://doi.org/10.1016/0891-5849(94)90019-1.

(77) Buettner, G. R.; Jurkiewicz, B. A. Catalytic Metals, Ascorbate and Free Radicals: Combinations to Avoid. *Radiat. Res.* **1996**, *145* (5), 532. https://doi.org/10.2307/3579271.

(78) Alfassi, Z. B.; Huie, R. E.; Neta, P.; Shoute, L. C. T. Temperature Dependence of the Rate Constants for Reaction of Inorganic Radicals with Organic Reductants. *J. Phys. Chem.* **1990**, *94* (25), 8800–8805. https://doi.org/10.1021/j100388a011.

(79) Augusto, O.; Bonini, M. G.; Amanso, A. M.; Linares, E.; Santos, C. C. X.; De Menezes, S. L. Nitrogen Dioxide and Carbonate Radical Anion: Two Emerging Radicals in Biology. *Free Radic. Biol. Med.* **2002**, *32* (9), 841–859. https://doi.org/10.1016/S0891-5849(02)00786-4.

(80) Goldstein, S.; Czapski, G. Reactivity of Peroxynitrite versus Simultaneous Generation of $^{\bullet}NO$ and $O_2^{\bullet -}$ toward NADH. *Chem. Res. Toxicol.* **2000**, *13* (8), 736–741. https://doi.org/10.1021/tx000099n.

(81) Graetzel, M. Pulsradiolytische Untersuchung einiger Elementarprozesse der Oxydation und Reduktion des Nitritions. *Berichte Bunsenges. Fuer Phys. Chem.* **1969**, *73* (7).

(82) Jacob, D. Heterogeneous Chemistry and Tropospheric Ozone. *Atmos. Environ.* **2000**, *34* (12–14), 2131–2159. https://doi.org/10.1016/S1352-2310(99)00462-8.

(83) Bonini, M. G.; Augusto, O. Carbon Dioxide Stimulates the Production of Thiyl, Sulfinyl, and Disulfide Radical Anion from Thiol Oxidation by Peroxynitrite. *J. Biol. Chem.* **2001**, *276* (13), 9749–9754. https://doi.org/10.1074/jbc.M008456200.

(84) Kurz, C. R.; Kissner, R.; Nauser, T.; Perrin, D.; Koppenol, W. H. Rapid Scavenging of Peroxynitrous Acid by Monohydroascorbate. *Free Radic. Biol. Med.* **2003**, *35* (12), 1529–1537. https://doi.org/10.1016/j.freeradbiomed.2003.08.012.

(85) Squadrito, G. L.; Cueto, R.; Splenser, A. E.; Valavanidis, A.; Zhang, H.; Uppu, R. M.; Pryor, W. A. Reaction of Uric Acid with Peroxynitrite and Implications for the Mechanism of Neuroprotection by Uric Acid. *Arch. Biochem. Biophys.* **2000**, *376* (2), 333–337. https://doi.org/10.1006/abbi.2000.1721.

(86) Arana, A. A.; Artaxo, P.; Rizzo, L. V.; Bastos, W. Long Term Measurements of the Elemental Composition and Optical Properties of Aerosols in Amazonia. *E3S Web Conf.* **2013**, *1*, 03005. https://doi.org/10.1051/e3sconf/20130103005.

(87) Birmili, W.; Allen, A. G.; Bary, F.; Harrison, R. M. Trace Metal Concentrations and Water Solubility in Size-Fractionated Atmospheric Particles and Influence of Road Traffic. *Environ. Sci. Technol.* **2006**, *40* (4), 1144–1153. https://doi.org/10.1021/es0486925.

(88) Heal, M. R.; Hibbs, L. R.; Agius, R. M.; Beverland, I. J. Total and Water-Soluble Trace Metal Content of Urban Background PM10, PM2.5 and Black Smoke in Edinburgh, UK. *Atmos. Environ.* **2005**, *39* (8), 1417–1430. https://doi.org/10.1016/j.atmosenv.2004.11.026.

(89) Harrison, R. M.; Yin, J. Chemical Speciation of PM2.5 Particles at Urban Background and Rural Sites in the UK Atmosphere. *J. Environ. Monit.* **2010**, *12* (7), 1404–1414. https://doi.org/10.1039/c000329h.

(90) Maenhaut, W.; Salma, I.; Cafmeyer, J.; Annegarn, H. J.; Andreae, M. O. Regional Atmospheric Aerosol Composition and Sources in the Eastern Transvaal, South Africa, and Impact of Biomass Burning. *J. Geophys. Res. Atmospheres* **1996**, *101* (D19), 23631–23650. https://doi.org/10.1029/95JD02930.

(91) Artaxo, P.; Gerab, F.; Yamasoe, M. A.; Martins, J. V. Fine Mode Aerosol Composition at Three Long-Term Atmospheric Monitoring Sites in the Amazon Basin. *J. Geophys. Res.* **1994**, *99* (D11), 22857–22868. https://doi.org/10.1029/94JD01023.

(92) Pakkanen, T. A.; Loukkola, K.; Korhonen, C. H.; Aurela, M.; Mäkelä, T.; Hillamo, R. E.; Aarnio, P.; Koskentalo, T.; Kousa, A.; Maenhaut, W. Sources and Chemical Composition of Atmospheric Fine and Coarse Particles in the Helsinki Area. *Atmos. Environ.* **2001**, *35* (32), 5381–5391. https://doi.org/10.1016/S1352-2310(01)00307-7.

(93) Olson, D. A.; Turlington, J.; Duvall, R. M.; McDow, S. R.; Stevens, C. D.; Williams, R. Indoor and Outdoor Concentrations of Organic and Inorganic Molecular Markers: Source Apportionment of PM2.5 Using Low-Volume Samples. *Atmos. Environ.* **2008**, *42* (8), 1742–1751. https://doi.org/10.1016/j.atmosenv.2007.11.035.

(94) Lee, P. K. H.; Brook, J. R.; Dabek-Zlotorzynska, E.; Mabury, S. A. Identification of the Major Sources Contributing to $PM_{2.5}$ Observed in Toronto. *Environ. Sci. Technol.* **2003**, *37* (21), 4831–4840. https://doi.org/10.1021/es026473i.

(95) Upadhyay, N.; Clements, A.; Fraser, M.; Herckes, P. Chemical Speciation of $PM_{2.5}$ and PM_{10} in South Phoenix, AZ. *J. Air Waste Manag. Assoc.* **2011**, *61* (3), 302–310. https://doi.org/10.3155/1047-3289.61.3.302.

(96) Hassanvand, M. S.; Naddafi, K.; Faridi, S.; Nabizadeh, R.; Sowlat, M. H.; Momeniha, F.; Gholampour, A.; Arhami, M.; Kashani, H.; Zare, A.; Niazi, S.; Rastkari, N.; Nazmara, S.; Ghani, M.; Yunesian, M. Characterization of PAHs and Metals in Indoor/Outdoor PM10/PM2.5/PM1 in a Retirement Home and a School Dormitory. *Sci. Total Environ.* **2015**, *527–528*, 100–110. https://doi.org/10.1016/j.scitotenv.2015.05.001.

(97) Contini, D.; Cesari, D.; Donateo, A.; Chirizzi, D.; Belosi, F. Characterization of PM10 and PM2.5 and Their Metals Content in Different Typologies of Sites in South-Eastern Italy. *Atmosphere* **2014**, *5* (2), 435–453. https://doi.org/10.3390/atmos5020435.

(98) Manousakas, M.; Papaefthymiou, H.; Eleftheriadis, K.; Katsanou, K. Determination of Water-Soluble and Insoluble Elements in PM2.5 by ICP-MS. *Sci. Total Environ.* **2014**, *493*, 694–700. https://doi.org/10.1016/j.scitotenv.2014.06.043.

(99) Han, Y.-J.; Kim, H.-W.; Cho, S.-H.; Kim, P.-R.; Kim, W.-J. Metallic Elements in PM2.5 in Different Functional Areas of Korea: Concentrations and Source Identification. *Atmospheric Res.* **2015**, *153*, 416–428. https://doi.org/10.1016/j.atmosres.2014.10.002.

(100) Maenhaut, W.; Raes, N.; Chi, X.; Cafmeyer, J.; Wang, W.; Salma, I. Chemical Composition and Mass Closure for Fine and Coarse Aerosols at a Kerbside in Budapest, Hungary, in Spring 2002. *X-Ray Spectrom.* **2005**, *34* (4), 290–296. https://doi.org/10.1002/xrs.820.

(101) Rogula-Kozłowska, W.; Błaszczak, B.; Szopa, S.; Klejnowski, K.; Sówka, I.; Zwoździak, A.; Jabłońska, M.; Mathews, B. PM2.5 in the Central Part of Upper Silesia, Poland: Concentrations, Elemental Composition, and Mobility of Components. *Environ. Monit. Assess.* **2013**, *185* (1), 581–601. https://doi.org/10.1007/s10661-012-2577-1.

(102) Morishita, M.; Keeler, G. J.; Kamal, A. S.; Wagner, J. G.; Harkema, J. R.; Rohr, A. C. Identification of Ambient PM2.5 Sources and Analysis of Pollution Episodes in Detroit, Michigan Using Highly Time-Resolved Measurements. *Atmos. Environ.* **2011**, *45* (8), 1627–1637. https://doi.org/10.1016/j.atmosenv.2010.09.062.

(103) Chow, J. C.; Watson, J. G.; Fujita, E. M.; Lu, Z.; Lawson, D. R.; Ashbaugh, L. L. Temporal and Spatial Variations of PM2.5 and PM10 Aerosol in the Southern California Air Quality Study. *Atmos. Environ.* **1994**, *28* (12), 2061–2080. https://doi.org/10.1016/1352-2310(94)90474-X.

(104) Vecchi, R.; Marcazzan, G.; Valli, G.; Ceriani, M.; Antoniazzi, C. The Role of Atmospheric Dispersion in the Seasonal Variation of PM1 and PM2.5 Concentration and Composition in the Urban Area of Milan (Italy). *Atmos. Environ.* **2004**, *38* (27), 4437–4446. https://doi.org/10.1016/j.atmosenv.2004.05.029.

(105) Khodeir, M.; Shamy, M.; Alghamdi, M.; Zhong, M.; Sun, H.; Costa, M.; Chen, L.-C.; Maciejczyk, P. Source Apportionment and Elemental Composition of PM2.5 and PM10 in Jeddah City, Saudi Arabia. *Atmospheric Pollut. Res.* **2012**, *3* (3), 331–340. https://doi.org/10.5094/APR.2012.037.

(106) Hagler, G. S. W.; Bergin, M. H.; Salmon, L. G.; Yu, J. Z.; Wan, E. C. H.; Zheng, M.; Zeng, L. M.; Kiang, C. S.; Zhang, Y. H.; Schauer, J. J. Local and Regional Anthropogenic Influence on PM2.5 Elements in Hong Kong. *Atmos. Environ.* **2007**, *41* (28), 5994–6004. https://doi.org/10.1016/j.atmosenv.2007.03.012.

(107) Loyola, J.; Arbilla, G.; Quiterio, S. L.; Escaleira, V.; Minho, A. S. Trace Metals in the Urban Aerosols of Rio de Janeiro City. *J. Braz. Chem. Soc.* **2012**, *23* (4), 628–638. https://doi.org/10.1590/S0103-50532012000400007.

(108) Squizzato, S.; Masiol, M.; Visin, F.; Canal, A.; Rampazzo, G.; Pavoni, B. The PM2.5 Chemical Composition in an Industrial Zone Included in a Large Urban Settlement: Main Sources and Local Background. *Environ. Sci. Process. Impacts* **2014**, *16* (8), 1913–1922. https://doi.org/10.1039/C4EM00111G.

(109) Matschullat, J.; Maenhaut, W.; Zimmermann, F.; Juliane Fiebig. Aerosol and Bulk Deposition Trends in the 1990's, Eastern Erzgebirge, Central Europe. *Atmos. Environ.* **2000**, *34* (19), 3213–3221. https://doi.org/10.1016/S1352-2310(99)00516-6.

(110) Querol, X.; Alastuey, A.; Rodriguez, S.; Plana, F.; Ruiz, C. R.; Cots, N.; Massagué, G.; Puig, O. PM10 and PM2.5 Source Apportionment in the Barcelona Metropolitan Area, Catalonia, Spain. *Atmos. Environ.* **2001**, *35* (36), 6407–6419. https://doi.org/10.1016/S1352-2310(01)00361-2.

(111) Martinez, M. A.; Caballero, P.; Carrillo, O.; Mendoza, A.; Mejia, G. M. Chemical Characterization and Factor Analysis of $PM_{2.5}$ in Two Sites of Monterrey, Mexico. *J. Air Waste Manag. Assoc.* **2012**, *62* (7), 817–827. https://doi.org/10.1080/10962247.2012.681421.

(112) Janssen, N. A. H.; Van Mansom, D. F. M.; Van Der Jagt, K.; Harssema, H.; Hoek, G. Mass Concentration and Elemental Composition of Airborne Particulate Matter at Street and Background Locations. *Atmos. Environ.* **1997**, *31* (8), 1185–1193. https://doi.org/10.1016/S1352-2310(96)00291-9.

(113) Na, K.; Cocker, D. R. Characterization and Source Identification of Trace Elements in PM2.5 from Mira Loma, Southern California. *Atmospheric Res.* **2009**, *93* (4), 793–800. https://doi.org/10.1016/j.atmosres.2009.03.012.

(114) Shaltout, A. A.; Boman, J.; Al-Malawi, D. R.; Shehadeh, Z. F. Elemental Composition of PM2.5 Particles Sampled in Industrial and Residential Areas of Taif, Saudi Arabia. *Aerosol Air Qual. Res.* **2013**, *13* (4), 1356–1364. https://doi.org/10.4209/aaqr.2012.11.0320.

(115) Chow, J. C.; Watson, J. G.; Lu, Z.; Lowenthal, D. H.; Frazier, C. A.; Solomon, P. A.; Thuillier, R. H.; Magliano, K. Descriptive Analysis of PM2. 5 and PM10 at Regionally Representative Locations during SJVAQS/AUSPEX. *Atmos. Environ.* **1996**, *30* (12), 2079–2112. https://doi.org/10.1016/1352-2310(95)00402-5.

(116) Kendall, M.; Pala, K.; Ucakli, S.; Gucer, S. Airborne Particulate Matter (PM2.5 and PM10) and Associated Metals in Urban Turkey. *Air Qual. Atmosphere Health* **2011**, *4* (3–4), 235–242. https://doi.org/10.1007/s11869-010-0129-9.

(117) Mansha, M.; Ghauri, B.; Rahman, S.; Amman, A. Characterization and Source Apportionment of Ambient Air Particulate Matter (PM2.5) in Karachi. *Sci. Total Environ.* **2012**, *425*, 176–183. https://doi.org/10.1016/j.scitotenv.2011.10.056.

(118) Pant, P.; Shukla, A.; Kohl, S. D.; Chow, J. C.; Watson, J. G.; Harrison, R. M. Characterization of Ambient PM2.5 at a Pollution Hotspot in New Delhi, India and Inference of Sources. *Atmos. Environ.* **2015**, *109*, 178–189. https://doi.org/10.1016/j.atmosenv.2015.02.074.

(119) Tolis, E. I.; Saraga, D. E.; Filiou, K. F.; Tziavos, N. I.; Tsiaousis, C. P.; Dinas, A.; Bartzis, J. G. One-Year Intensive Characterization on PM2.5 Nearby Port Area of Thessaloniki, Greece. *Environ. Sci. Pollut. Res.* **2015**, *22* (9), 6812–6826. https://doi.org/10.1007/s11356-014-3883-7.

(120) López, M. L.; Ceppi, S.; Palancar, G. G.; Olcese, L. E.; Tirao, G.; Toselli, B. M. Elemental Concentration and Source Identification of PM10 and PM2.5 by SR-XRF in Córdoba City, Argentina. *Atmos. Environ.* **2011**, *45* (31), 5450–5457. https://doi.org/10.1016/j.atmosenv.2011.07.003.

(121) Cao, L.; Zeng, J.; Liu, K.; Bao, L.; Li, Y. Characterization and Cytotoxicity of PM<0.2, PM0.2–2.5 and PM2.5–10 around MSWI in Shanghai, China. *Int. J. Environ. Res. Public. Health* **2015**, *12* (5), 5076–5089. https://doi.org/10.3390/ijerph120505076.

(122) Yin, L.; Niu, Z.; Chen, X.; Chen, J.; Xu, L.; Zhang, F. Chemical Compositions of PM2.5 Aerosol during Haze Periods in the Mountainous City of Yong'an, China. *J. Environ. Sci.* **2012**, *24* (7), 1225–1233. https://doi.org/10.1016/S1001-0742(11)60940-6.

(123) Zhou, S.; Yuan, Q.; Li, W.; Lu, Y.; Zhang, Y.; Wang, W. Trace Metals in Atmospheric Fine Particles in One Industrial Urban City: Spatial Variations, Sources, and Health Implications. *J. Environ. Sci.* **2014**, *26* (1), 205–213. https://doi.org/10.1016/S1001-0742(13)60399-X.

(124) Wang, X.; Bi, X.; Sheng, G.; Fu, J. Chemical Composition and Sources of PM10 and PM2.5 Aerosols in Guangzhou, China. *Environ. Monit. Assess.* **2006**, *119* (1–3), 425–439. https://doi.org/10.1007/s10661-005-9034-3.

(125) Kulshrestha, A.; Satsangi, P. G.; Masih, J.; Taneja, A. Metal Concentration of PM2.5 and PM10 Particles and Seasonal Variations in Urban and Rural Environment of Agra, India. *Sci. Total Environ.* **2009**, *407* (24), 6196–6204. https://doi.org/10.1016/j.scitotenv.2009.08.050.

(126) Yadav, S.; Satsangi, P. G. Characterization of Particulate Matter and Its Related Metal Toxicity in an Urban Location in South West India. *Environ. Monit. Assess.* **2013**, *185* (9), 7365–7379. https://doi.org/10.1007/s10661-013-3106-6.

(127) Song, S.; Wu, Y.; Jiang, J.; Yang, L.; Cheng, Y.; Hao, J. Chemical Characteristics of Size-Resolved PM2.5 at a Roadside Environment in Beijing, China. *Environ. Pollut.* **2012**, *161*, 215–221. https://doi.org/10.1016/j.envpol.2011.10.014.

(128) Sun, Y.; Zhuang, G.; Wang, Y.; Han, L.; Guo, J.; Dan, M.; Zhang, W.; Wang, Z.; Hao, Z. The Air-Borne Particulate Pollution in Beijing—Concentration, Composition, Distribution and Sources. *Atmos. Environ.* **2004**, *38* (35), 5991–6004. https://doi.org/10.1016/j.atmosenv.2004.07.009.

(129) See, S. W.; Balasubramanian, R.; Rianawati, E.; Karthikeyan, S.; Streets, D. G. Characterization and Source Apportionment of Particulate Matter ≤ 2.5 Mm in Sumatra, Indonesia, during a Recent Peat Fire Episode. *Environ. Sci. Technol.* **2007**, *41* (10), 3488–3494. https://doi.org/10.1021/es061943k.

(130) Poschl, U.; Martin, S. T.; Sinha, B.; Chen, Q.; Gunthe, S. S.; Huffman, J. A.; Borrmann, S.; Farmer, D. K.; Garland, R. M.; Helas, G.; Jimenez, J. L.; King, S. M.; Manzi, A.; Mikhailov, E.; Pauliquevis, T.; Petters, M. D.; Prenni, A. J.; Roldin, P.; Rose, D.; Schneider, J.; Su, H.; Zorn, S. R.; Artaxo, P.; Andreae, M. O. Rainforest Aerosols as Biogenic Nuclei of Clouds and Precipitation in the Amazon. *Science* **2010**, *329* (5998), 1513–1516. https://doi.org/10.1126/science.1191056.

(131) Jimenez, J. L.; Canagaratna, M. R.; Donahue, N. M.; Prevot, A. S. H.; Zhang, Q.; Kroll, J. H.; DeCarlo, P. F.; Allan, J. D.; Coe, H.; Ng, N. L.; Aiken, A. C.; Docherty, K. S.; Ulbrich, I. M.; Grieshop, A. P.; Robinson, A. L.; Duplissy, J.; Smith, J. D.; Wilson, K. R.; Lanz, V. A.; Hueglin, C.; Sun, Y. L.; Tian, J.; Laaksonen, A.; Raatikainen, T.; Rautiainen, J.; Vaattovaara, P.; Ehn, M.; Kulmala, M.; Tomlinson, J. M.; Collins, D. R.; Cubison, M. J.; E.; Dunlea, J.; Huffman, J. A.; Onasch, T. B.; Alfarra, M. R.; Williams, P. I.; Bower, K.; Kondo, Y.; Schneider, J.; Drewnick, F.; Borrmann, S.; Weimer, S.; Demerjian, K.; Salcedo, D.; Cottrell, L.; Griffin, R.; Takami, A.; Miyoshi, T.; Hatakeyama, S.; Shimono, A.; Sun, J. Y.; Zhang, Y. M.; Dzepina, K.; Kimmel, J. R.; Sueper, D.; Jayne, J. T.; Herndon, S. C.; Trimborn, A. M.; Williams, L. R.; Wood, E. C.; Middlebrook, A. M.; Kolb, C. E.; Baltensperger, U.; Worsnop, D. R. Evolution of Organic Aerosols in the Atmosphere. *Science* **2009**, *326* (5959), 1525–1529. https://doi.org/10.1126/science.1180353.

(132) Huang, R.-J.; Zhang, Y.; Bozzetti, C.; Ho, K.-F.; Cao, J.-J.; Han, Y.; Daellenbach, K. R.; Slowik, J. G.; Platt, S. M.; Canonaco, F.; Zotter, P.; Wolf, R.; Pieber, S. M.; Bruns, E. A.; Crippa, M.; Ciarelli, G.; Piazzalunga, A.; Schwikowski, M.; Abbaszade, G.; Schnelle-Kreis, J.; Zimmermann, R.; An, Z.; Szidat, S.; Baltensperger, U.; Haddad, I. E.; Prévôt, A. S. H. High Secondary Aerosol Contribution to Particulate Pollution during Haze Events in China. *Nature* **2014**, *514* (7521), 218–222. https://doi.org/10.1038/nature13774.

(133) Wingfors, H. Characterization of the Size-Distribution of Aerosols and Particle-Bound Content of Oxygenated PAHs, PAHs, and n-Alkanes in Urban Environments in Afghanistan. *Atmos. Environ.* **2011**, *45* (26), 4360–4369. https://doi.org/10.1016/j.atmosenv.2011.05.049.

(134) Valavanidis, A.; Fiotakis, K.; Vlahogianni, T.; Papadimitriou, V.; Pantikaki, V. Determination of Selective Quinones and Quinoid Radicals in Airborne Particulate Matter and Vehicular Exhaust Particles. *Environ. Chem.* **2006**, *3* (2), 118–123. https://doi.org/10.1071/EN05089.

(135) Eiguren-Fernandez, A.; Miguel, A. H.; Di Stefano, E.; Schmitz, D. A.; Cho, A. K.; Thurairatnam, S.; Avol, E. L.; Froines, J. R. Atmospheric Distribution of Gas- and Particle-Phase Quinones in Southern California. *Aerosol Sci. Technol.* **2008**, *42* (10), 854–861. https://doi.org/10.1080/02786820802339546.

(136) Delgado-Saborit, J. M.; Alam, M. S.; Godri Pollitt, K. J.; Stark, C.; Harrison, R. M. Analysis of Atmospheric Concentrations of Quinones and Polycyclic Aromatic Hydrocarbons in Vapour and Particulate Phases. *Atmos. Environ.* **2013**, *77*, 974–982. https://doi.org/10.1016/j.atmosenv.2013.05.080.

(137) Alam, M. S.; Delgado-Saborit, J. M.; Stark, C.; Harrison, R. M. Investigating PAH Relative Reactivity Using Congener Profiles, Quinone Measurements and Back Trajectories. *Atmospheric Chem. Phys.* **2014**, *14* (5), 2467–2477. https://doi.org/10.5194/acp-14-2467-2014.

(138) Cho, A. K.; Di Stefano, E.; You, Y.; Rodriguez, C. E.; Schmitz, D. A.; Kumagai, Y.; Miguel, A. H.; Eiguren-Fernandez, A.; Kobayashi, T.; Avol, E.; Froines, J. R. Determination of Four Quinones in Diesel Exhaust Particles, SRM 1649a, and Atmospheric PM $_{2.5}$ Special Issue of *Aerosol Science and Technology* on Findings from the Fine Particulate Matter Supersites Program. *Aerosol Sci. Technol.* **2004**, *38* (sup1), 68–81. https://doi.org/10.1080/02786820390229471.

Table S1. Chemical reactions, rate constants as used in the KM-SUB-ELF, with reference

**#**	**Reaction**	**Rate constant (cm^{-3} s^{-1} or s^{-1})**	**Reference**
	Gas-phase reactions		
1	$NO^{\bullet} + O_3 \rightarrow NO_2^{\bullet} + O_2$	2.05×10^{-14}	[45,46]
2	$NO_2^{\bullet} + O_3 \rightarrow NO_3^{\bullet} + O_2$	4.85×10^{-17}	[45,46]
3	$NO^{\bullet} + NO^{\bullet} + O_2 \rightarrow NO_2^{\bullet} + NO_2^{\bullet}$	8.93×10^{-20}	[45,46]
4	$NO^{\bullet} + NO_3^{\bullet} \rightarrow NO_2^{\bullet} + NO_2^{\bullet}$	2.57×10^{-11}	[45,46]
5	$NO_2^{\bullet} + NO_3^{\bullet} \rightarrow NO^{\bullet} + NO_2^{\bullet} + O_2$	7.73×10^{-16}	[45,46]
6	$NO_2^{\bullet} + NO_3^{\bullet} \rightarrow N_2O_5$	1.21×10^{-12}	[45,46]
7	$^{\bullet}OH + O_3 \rightarrow HO_2^{\bullet} + O_2$	8.20×10^{-14}	[45,46]
8	$^{\bullet}OH + H_2O_2 \rightarrow HO_2^{\bullet} + H_2O$	1.73×10^{-12}	[45,46]
9	$HO_2^{\bullet} + O_3 \rightarrow {}^{\bullet}OH + O_2 + O_2$	8.24×10^{-16}	[45,46]
10	$^{\bullet}OH + HO_2^{\bullet} \rightarrow H_2O + O_2$	1.08×10^{-10}	[45,46]
11	$HO_2^{\bullet} + HO_2^{\bullet} \rightarrow H_2O_2 + O_2$	5.09×10^{-12}	[45,46]
12	$HO_2^{\bullet} + HO_2^{\bullet} \rightarrow H_2O_2$	3.50×10^{-12}	[45,46]
13	$^{\bullet}OH + NO^{\bullet} \rightarrow HONO$	8.91×10^{-12}	[45,46]
14	$^{\bullet}OH + NO_2^{\bullet} \rightarrow HNO_3$	8.91×10^{-12}	[45,46]
15	$^{\bullet}OH + NO_3^{\bullet} \rightarrow HO_2^{\bullet} + NO_2^{\bullet}$	2.00×10^{-11}	[45,46]
16	$HO_2^{\bullet} + NO^{\bullet} \rightarrow {}^{\bullet}OH + NO_2^{\bullet}$	8.24×10^{-12}	[45,46]
17	$HO_2^{\bullet} + NO_2^{\bullet} \rightarrow HO_2NO_2$	6.87×10^{-13}	[45,46]
18	$HO_2NO_2 \rightarrow HO_2^{\bullet} + NO_2^{\bullet}$	2.49×10^{-1}	[45,46]
19	$^{\bullet}OH + HO_2NO_2 \rightarrow NO_2^{\bullet} + H_2O + O_2$	2.96×10^{-12}	[45,46]
20	$HO_2^{\bullet} + NO_3^{\bullet} \rightarrow {}^{\bullet}OH + NO_2^{\bullet}$	4.00×10^{-12}	[45,46]
21	$^{\bullet}OH + HONO \rightarrow NO_2^{\bullet} + H_2O$	5.78×10^{-12}	[45,46]
22	$^{\bullet}OH + HNO_3 \rightarrow NO_3^{\bullet} + H_2O$	1.37×10^{-13}	[45,46]
23	$N_2O_5 \rightarrow NO_2^{\bullet} + NO_3^{\bullet}$	1.83×10^{-1}	[45,46]
	Surfactant reactions		
24	SPB + $^{\bullet}OH$ → SPB-ox	1.70×10^{-11}	[47–49]
25	POG + $^{\bullet}OH$ → POG-ox	1.70×10^{-11}	[1]
26	SPB + O_3 → SPB-ox	1.00×10^{-14}	[50,51]
27	POG + O_3 → POG-ox + 0.17 H_2O_2	1.66×10^{-16}	[52–55]
28	aToc + OH → aToc-ox	4.50×10^{-13}	[56]
29	aToc + O_3 → aToc-ox	1.20×10^{-18}	[57]
	ELF reactions		
30	$O_2^{\bullet-} + HO_2 + H_2O \rightarrow H_2O_2 + OH^- + O_2$	1.70×10^{-13}	[1,58]
31	$HO_2 + HO_2 \rightarrow H_2O_2 + O_2$	1.40×10^{-15}	[58]

32	$O_2^{\bullet-} + O_2^{\bullet-} + 2H^+ \rightarrow H_2O_2 + O_2$	3.82×10^{-16}	[58]
33	$H_2O_2 + {}^{\bullet}OH \rightarrow HO_2 + H_2O$	5.50×10^{-14}	[59]
34	${}^{\bullet}OH + {}^{\bullet}OH \rightarrow H_2O_2$	8.60×10^{-12}	[60]
35	${}^{\bullet}OH + O_2^{\bullet-} \rightarrow O_2 + OH^-$	1.30×10^{-11}	[47]
36	${}^{\bullet}OH + HO_2 \rightarrow H_2O + O_2$	1.20×10^{-11}	[60]
37	$H_2O_2 + HO_2 \rightarrow {}^{\bullet}OH + O_2 + H_2O$	4.98×10^{-21}	[61]
38	$Fe^{2+} + O_2^- + 2H^+ \rightarrow Fe^{3+} + H_2O_2$	3.10×10^{-14}	[1,58]
39	$Fe^{2+} + HO_2 + H^+ \rightarrow Fe^{3+} + H_2O_2$	1.99×10^{-15}	[62]
40	$Fe^{2+} + H_2O_2 \rightarrow Fe^{3+} + {}^{\bullet}OH + OH^-$	4.30×10^{-18}	[63]
41	$Fe^{2+} + {}^{\bullet}OH \rightarrow Fe^{3+} + OH^-$	5.30×10^{-13}	[64]
42	$Fe^{2+} + H_2O_2 \rightarrow Fe^{4+} + H_2O$	9.50×10^{-18}	[1]
43	$Fe^{3+} + H_2O_2 \rightarrow Fe^{2+} + HO_2 + H^+$	3.32×10^{-24}	[63]
44	$Fe^{3+} + HO_2 \rightarrow Fe^{2+} + O_2 + H^+$	3.30×10^{-18}	[58]
45	$Fe^{4+} + Fe^{2+} \rightarrow Fe^{3+} + Fe^{3+}$	6.60×10^{-18}	[41]
46	$Fe^{3+} + AscH \rightarrow Fe^{2+} + Asc^{\bullet}$	1.10×10^{-19}	[1]
47	$Fe^{4+} + AscH \rightarrow Fe^{3+} + Asc^{\bullet}$	7.60×10^{-19}	[1]
48	$Fe^{2+} + O_2 \rightarrow O_2^{\bullet-} + Fe^{3+}$	5.20×10^{-21}	[1]
49	$Cu^+ + HO_2 + H^+ \rightarrow Cu^{2+} + H_2O_2$	2.30×10^{-12}	[1]
50	$Cu^+ + O_2^{\bullet-} + H_2O \rightarrow Cu^{2+} + H_2O_2 + OH^-$	5.80×10^{-15}	[1]
51	$Cu^{2+} + HO_2 \rightarrow Cu^+ + O_2 + H^+$	1.60×10^{-11}	[1]
52	$Cu^{2+} + O_2^{\bullet-} \rightarrow Cu^+ + O_2$	8.30×10^{-12}	[1]
53	$Cu^{2+} + AscH \rightarrow Cu^+ + Asc^{\bullet}$	1.40×10^{-18}	[1]
54	$Cu^+ + O_2 \rightarrow Cu^{2+} + O_2^{\bullet-}$	6.90×10^{-20}	[1]
55	$Cu^+ + H_2O_2 \rightarrow Cu^{2+} + {}^{\bullet}OH + OH^-$	2.40×10^{-20}	[1]
56	$Cu^+ + H_2O_2 \rightarrow Cu^{3+} + OH^- + OH^-$	5.00×10^{-19}	[1]
57	$Cu^+ + Cu^{3+} \rightarrow Cu^{2+} + Cu^{2+}$	5.80×10^{-12}	[1]
58	$Cu^{2+} + H_2O_2 \rightarrow Cu^+ + O_2^{\bullet-} + H^+$	3.80×10^{-24}	[1]
59	$PQN + AscH \rightarrow PQN^{\bullet} + Asc^{\bullet}$	1.20×10^{-20}	[29]
60	$PQN^{\bullet} + O_2 \rightarrow PQN + O_2^{\bullet-}$	4.60×10^{-13}	[1]
61	$PQN^{\bullet} + O_2^{\bullet-} + 2H^+ \rightarrow PQN + H_2O_2$	3.30×10^{-12}	[1]
62	$NQN12 + AscH \rightarrow NQN12^{\bullet} + Asc^{\bullet}$	1.50×10^{-19}	[29]
63	$NQN12^{\bullet} + O_2 \rightarrow NQN12 + O_2^{\bullet-}$	4.60×10^{-13}	[1]
64	$NQN12^{\bullet} + O_2^{\bullet-} + 2H^+ \rightarrow NQN12 + H_2O_2$	3.30×10^{-12}	[1]
65	$NQN14 + AscH \rightarrow NQN14^{\bullet} + Asc^{\bullet}$	6.30×10^{-21}	[29]
66	$NQN14^{\bullet} + O_2 \rightarrow NQN14 + O_2^{\bullet-}$	4.60×10^{-13}	[1]
67	$NQN14^{\bullet} + O_2^{\bullet-} + 2H^+ \rightarrow NQN14 + H_2O_2$	3.30×10^{-12}	[1]
68	$UA + O_3 \rightarrow$ Products	9.60×10^{-17}	[57]

69	$UA + {}^{\bullet}OH \rightarrow Products + OH^-$	1.20×10^{-11}	[65]
70	$GSH + {}^{\bullet}OH \rightarrow Products + OH^-$	1.50×10^{-11}	[66]
71	$GSSG + {}^{\bullet}OH \rightarrow Products + OH^-$	1.50×10^{-11}	Assumed to be the same as R67
72	$Asc^{\bullet} + Asc^{\bullet} + H^+ \rightarrow AscH + DHA$	5.00×10^{-16}	[67]
73	$AscH + O_2^{\bullet -} + H^+ \rightarrow Asc^{\bullet} + H_2O_2$	5.10×10^{-17}	[1]
74	$AscH + HO_2 \rightarrow Asc^{\bullet} + H_2O_2$	2.65×10^{-17}	[68]
75	$AscH + {}^{\bullet}OH \rightarrow Products + OH^-$	1.80×10^{-11}	[69]
76	$AscH + {}^{\bullet}O_3 \rightarrow Products$	9.10×10^{-17}	[57]
77	$1.25\ GS^- + 0.5\ O_3 \rightarrow Products$	9.60×10^{-20}	[57]
78	$1.25\ GSH + 0.5\ O_3 \rightarrow Products$	9.60×10^{-20}	[57]
79	$GSOO + GSOO \rightarrow 0.56\ O_2^{\bullet -} + Products$	6.79×10^{-13}	[70]
80	$O_2^{\bullet -} + GSH \rightarrow GSO^{\bullet} + OH^-$	3.32×10^{-19}	[71–73]
81	$NO_2^{\bullet} + GS^{\bullet} \rightarrow GSNO_2$	4.98×10^{-12}	[4]
82	$GSOO^{\bullet} + NO_2^{\bullet} \rightarrow GSOONO_2$	2.49×10^{-12}	[70]
83	$GSOONO_2 \rightarrow GSOO^{\bullet} + NO_2^{\bullet}$	7.5×10^{-1}	[70]
84	$NO_2^{\bullet} + GS^- \rightarrow NO_2^- + GS^{\bullet}$	4.00×10^{-13}	[4]
85	$NO_2^{\bullet} + GSH \rightarrow NO_2^- + GS^{\bullet} + H^+$	1.66×10^{-14}	[74]
86	$GSOO^{\bullet} + GSH \rightarrow GSO^{\bullet} + GSOH$	3.32×10^{-15}	[4]
87	$GSO + NO_2 \rightarrow GSOONO$	7.47×10^{-12}	[4]
88	$GSOONO \rightarrow Products$	7.00×10^{2}	[4]
89	$GS^{\bullet} + GS^- \rightarrow GSSG^{\bullet -}$	1.59×10^{-14}	[4,5]
90	$GSSG^{\bullet -} \rightarrow GS^{\bullet} + GS^-$	1.60×10^{5}	[4,5]
91	$GSSG^{\bullet -} + O_2 \rightarrow GSSG + O_2^{\bullet -}$	8.30×10^{-12}	[4,5]
92	$GS^{\bullet} + GS^{\bullet} \rightarrow GSSG$	8.30×10^{-12}	[5]
93	$GSOH + GSH \rightarrow GSSG + H_2O$	1.20×10^{-18}	[75]
94	$GSO^{\bullet} + GSO^{\bullet} \rightarrow Products$	9.96×10^{-14}	[4]
95	$GS^- + H_2O_2 \rightarrow GSOH + OH^-$	1.60×10^{-21}	[75]
96	$GS^{\bullet} + AscH \rightarrow GSH + Asc^{\bullet}$	1.00×10^{-12}	[76,77]
97	$UA + NO_2^{\bullet} \rightarrow UA^{\bullet} + NO_2^-$	3.00×10^{-14}	[78,79]
98	$AscH + NO_2^{\bullet} \rightarrow Asc^{\bullet} + NO_2^-$	5.80×10^{-14}	[78,79]
99	$UA^{\bullet} + AscH \rightarrow UA + Asc^{\bullet}$	1.70×10^{-15}	[77]
100	$GS^{\bullet} + UA \rightarrow GSH + UA^{\bullet}$	5.00×10^{-14}	[74]
101	$O_2^{\bullet -} + NO_2^{\bullet} \rightarrow O_2NOOm$	7.50×10^{-12}	[4,80]
102	$O_2NOO^- \rightarrow NO_2^- + O_2$	7.00×10^{-1}	[4]
103	$O_2NOO^- \rightarrow O_2^{\bullet -} + NO_2^{\bullet}$	1.10×10^{0}	[4]
104	$NO_2^{\bullet} + NO_2^{\bullet} \rightarrow N_2O_4$	7.50×10^{-13}	[81]

105	$N_2O_4 \rightarrow NO_2^{\bullet} + NO_2^{\bullet}$	6.90×10^{3}	[81]
106	$N_2O_4 + H_2O \rightarrow NO_2^{-} + NO_3^{-} + 2H^{+}$	1.00×10^{3}	[4]
107	$O_2^{-} + O_3 + H_2O \rightarrow {}^{\bullet}OH + 2O_2 + OH^{-}$	2.50×10^{-12}	[82]
108	$HO_2 + O_3 \rightarrow {}^{\bullet}OH + 2O_2$	1.66×10^{-17}	[82]
109	$NO_2^{-} + {}^{\bullet}OH \rightarrow NO_2^{\bullet} + OH^{-}$	8.80×10^{-12}	[80]
110	${}^{\bullet}OH + NO_2^{\bullet} \rightarrow NO_3^{-} + H^{+}$	7.50×10^{-12}	[4]
111	${}^{\bullet}OH + NO_2^{\bullet} \rightarrow ONOOH$	7.50×10^{-12}	[4]
112	$ONOOH \rightarrow NO_2^{\bullet} + {}^{\bullet}OH$	3.00×10^{-1}	[4]
113	$ONOOH \rightarrow NO_3^{-} + H^{+}$	7.00×10^{-1}	[4]
114	$ONOO^{-} + GSH \rightarrow NO_2^{-} + GSOH$	1.10×10^{-18}	[83]
115	$GSO^{\bullet} + NO_2^{\bullet} \rightarrow GSOONO$	7.50×10^{-12}	[4]
116	$GSOONO + H_2O \rightarrow$ Products	7.00×10^{2}	[4]
117	$ONOOH + AscH \rightarrow Im_1$	1.66×10^{-15}	[84]
118	$Im_1 \rightarrow ONOOH + AscH$	5.00×10^{2}	[84]
119	$Im_1 \rightarrow Im_2$	4.00×10^{1}	[84]
120	$Im_2 \rightarrow Im_1$	5.00×10^{0}	[84]
121	$Im_2 + AscH \rightarrow Asc + DHA + NO_2^{-} + H_2O$	1.66×10^{-19}	[84]
122	$Im_2 \rightarrow Asc + NO_3^{-} + H^{+}$	8.50×10^{-1}	[84]
123	$ONOOH + UA \rightarrow UA^{rad} + NO_2$ + Products	2.60×10^{-19}	[85]
124	$O_2^{\bullet -} + SOD + H^{+} \rightarrow 0.5\ H_2O_2 + SOD$	2.65×10^{-12}	See text S2.
125	H_2O_2 + catalase $\rightarrow H_2O + 0.5\ O_2$ + catalase	3.20×10^{-14}	See text S2.
126	${}^{\bullet}OH$ + organic matter $\rightarrow$ oxidized organic matter	1.66×10^{-12}	[21,65]

Table S2. Input parameters to the KM-SUB-ELF model

Parameter	Description	Value
KH_{ccO3}	Henrys law equilibrium constant for O_3 [aq]/[gas]	1.7×10^{-1}
KH_{ccNO2}	Henrys law equilibrium constant for NO_2 [aq]/gas]	2.2×10^{-1}
KH_{ccNO}	Henrys law equilibrium constant for NO [aq]/[gas]	3.9×10^{-2}
KH_{ccOH}	Henrys law equilibrium constant for OH [aq]/[gas]	4.3×10^{2}
KH_{ccHO2}	Henrys law equilibrium constant for HO_2 [aq]/[gas]	1.7×10^{3}
KH_{ccH2O2}	Henrys law equilibrium constant for H_2O_2 [aq]/[gas]	9.9×10^{4}
ω_{O3}	Mean thermal velocity of O_3	3.7×10^{4} cm s^{-1}
ω_{NO2}	Mean thermal velocity of NO_2	3.8×10^{4} cm s^{-1}
ω_{NO}	Mean thermal velocity of NO	4.7×10^{4} cm s^{-1}
ω_{OH}	Mean thermal velocity of OH	6.2×10^{4} cm s^{-1}
ω_{HO2}	Mean thermal velocity of HO_2	4.5×10^{4} cm s^{-1}
ω_{H2O2}	Mean thermal velocity of H_2O_2	4.4×10^{4} cm s^{-1}
MW_{aa}	Average molecular weight of amino acids	125 g $mole^{-1}$
MW_{Cu}	Molecular weight copper	63.6 g $mole^{-1}$
MW_{Fe}	Molecular weight iron	55.6 g $mole^{-1}$
MW_{SOA}	Average molecular weight SOA	250 g $mole^{-1}$
MW_{PQN}	Molecular weight phenanthrenequinone (PQN)	208.2 g $mole^{-1}$
MW_{NQN}	Molecular weight naphthoquinone (NQN)	158.2 g $mole^{-1}$
pKa_{GSH}	Acid dissociation constant of GSH	8.8
pKa_{HO2}	Acid dissociation constant of HO_2	4.8
pKa_{ONOOH}	Acid dissociation constant of ONOOH	6.5
VR	Lung ventilation rate	1.5 m^3 h^{-1}
BR	Breathing rate	16 min^{-1}
FCR	Functional residual capacity of the lung	2750 cm^3
TV	Tidal Volume – Breath volume	1500 cm^3
SA_{ELF}	Total ELF surface area	890000 cm^2
T_{RT}	Respiratory tract temperature	37°C
pH_{ELF}	pH of the ELF	7

Table S3. List of symbols and definitions

Symbol	Meaning	SI unit
C_{Y}	ELF concentration of PM2.5 constituent Y	μmol L^{-1}
$C_{\mathrm{gas,PM2.5}}$	Ambient gas phase concentration of PM2.5	μg m^{-3}
$C_{\Sigma\mathrm{ROS}}$	ROS concentration in the ELF during or after model simulation	nmol L^{-1}
$d_{\mathrm{PM2.5}}$	Fraction of PM2.5 that deposits in the ELF	
$D_{\mathrm{PM2.5}}$	Dose of PM2.5 deposited in the ELF	μg
$I_{i\rightarrow \mathrm{j}}$	Gross chemical interconversion between individual ROS in the ELF	nmol L^{-1}
$I_{\Sigma\mathrm{ROS}}$	Gross chemical interconversion between all ROS in the ELF	nmol L^{-1}
MF_{Y}	PM2.5 mass fraction of constituent Y	
M_{Y}	Molar mass of PM2.5 constituent Y	g mol^{-1}
$N_{\Sigma\mathrm{ROS}}$	Cumulative production of ROS in the ELF	nmol L^{-1}
$N'_{\Sigma\mathrm{ROS}}$	Cumulative production rate of ROS in the ELF	nmol L^{-1} s^{-1}
$P_{\Sigma\mathrm{ROS}}$	Gross chemical production of ROS in the ELF	nmol L^{-1}
$P'_{\Sigma\mathrm{ROS}}$	Gross chemical production rate of ROS in the ELF	nmol L^{-1} s^{-1}
P	Gross chemical production of an individual ROS in the ELF	nmol L^{-1}
SF_{Y}	Soluble fraction of PM2.5 constituent Y in ELF	
t_{acc}	Accumulation time of PM2.5 in the ELF	h
t_{sim}	Model simulation time	h
V_{ELF}	Volume of the ELF	mL
VR	Lung ventilation rate	m^3 h^{-1}
Y_{OH}	OH yield in the ELF	pmol

**Table S4.** Mathematical formulas used to calculate ELF ROS metrics
$I_{\Sigma\mathrm{ROS}} = \Sigma_{\mathrm{j,i>j}}\ I_{i\rightarrow \mathrm{j}}$
$N_{\Sigma\mathrm{ROS}} = P_{\Sigma\mathrm{ROS}} - I_{\Sigma\mathrm{ROS}}$
$N'_{\Sigma\mathrm{ROS}} = (P_{\Sigma\mathrm{ROS}} - I_{\Sigma\mathrm{ROS}}) / t_{\mathrm{sim}}$
$P'_{\Sigma\mathrm{ROS}} = P_{\Sigma\mathrm{ROS}} / t_{\mathrm{sim}}$

Table S5. PM2.5 and transition metal concentrations with mass fractions as quantified in PM2.5 collected at different sampling sites throughout the world.

Sampling location	PM2.5 ($\mu g\ m^{-3}$)	Fe ($ng\ m^{-3}$)	Cu ($ng\ m^{-3}$)	Fe mass fraction	Cu mass fraction	Reference
Amazon (wet season)	1.65	33	0.07	2.00×10^{-2}	4.24×10^{-5}	[86]
Mace Head (Ireland)	4.2	5.68	0.71	1.35×10^{-3}	1.69×10^{-4}	[87]
Amazon (dry season)	4.87	19	0.8	3.90×10^{-3}	1.64×10^{-4}	[86]
Edinburgh (Scotland)	7.1	27.6	1.39	3.89×10^{-3}	1.96×10^{-4}	[88]
West Midlands (UK, rural)	7.6	51.3	13.5	6.75×10^{-3}	1.78×10^{-4}	[89]
West Midlands (UK, urban)	9	80.2	13.9	8.91×10^{-3}	1.54×10^{-3}	[89]
Skukuza (South Africa)	9.4	51	0.41	5.43×10^{-3}	4.36×10^{-5}	[90]
Amazon (Serro do Navio)	9.87	120	1.65	1.22×10^{-2}	1.67×10^{-4}	[91]
Amazon (Cuiabá)	10.5	175	1.55	1.67×10^{-2}	1.48×10^{-4}	[91]
West Midlands (UK, rural, average)	10.5	87.1	20	8.30×10^{-3}	1.91×10^{-3}	[89]
West Midlands (UK, urban, average)	11.6	102	21.9	8.79×10^{-3}	1.89×10^{-3}	[89]
Helsinki (Finland)	11.8	96	3.1	8.14×10^{-3}	2.63×10^{-4}	[92]
Tampa (Florida)	12.7	79	2.4	6.22×10^{-3}	1.89×10^{-4}	[93]
Toronto (Canada)	12.7	55	2.5	4.33×10^{-3}	1.97×10^{-4}	[94]
South Phoenix (Texas)	12.95	147	7.6	1.14×10^{-2}	5.87×10^{-4}	[95]
Tehran (inside a school dormitory)	14	102.13	22.17	7.30×10^{-3}	1.58×10^{-3}	[96]
Tehran (inside a retirement home)	15	130.78	25.1	8.72×10^{-3}	1.67×10^{-3}	[96]
South-Eastern Italy (background sites)	16.4	86.8	3.1	5.29×10^{-3}	1.89×10^{-4}	[97]
Patras (Greece)	17.4	124	7.28	7.13×10^{-3}	4.18×10^{-4}	[98]
Yeongwol (South Korea)	19.7	31.2	9.8	1.58×10^{-3}	4.98×10^{-4}	[99]
Budapest (Hungary)	20	430	18.3	2.15×10^{-2}	9.15×10^{-4}	[100]
South-Eastern Italy (industrial sites)	21.7	85	5.1	3.92×10^{-3}	2.35×10^{-4}	[97]

Zabrze (upper Silesia, Poland)	22	160.8	6.5	7.31×10^{-3}	2.96×10^{-4}	101
Chuncheon (South Korea)	23	29.6	9.9	1.29×10^{-3}	4.30×10^{-4}	99
Detroit (Michigan)	23	234	6	1.02×10^{-2}	2.61×10^{-4}	102
Megalopolis (Greece)	23	87	4.02	3.78×10^{-3}	1.75×10^{-4}	98
Tehran (outside a retirement home)	24	238.81	25.99	9.95×10^{-3}	1.08×10^{-3}	96
South-Eastern Italy (urban sites)	24.1	78.8	5.7	3.27×10^{-3}	2.37×10^{-4}	97
Tehran (outside a school dormitory)	26	280	32.42	1.08×10^{-2}	1.25×10^{-3}	96
Anaheim (California)	26.8	29.6	39.6	1.11×10^{-3}	1.48×10^{-3}	103
Milan (Summer, Italy)	27.2	186	10	6.84×10^{-3}	3.68×10^{-4}	104
Jeddah City (Saudi Arabia)	28.4	590	5.6	2.08×10^{-2}	1.97×10^{-4}	105
Hong Kong	29	140	5.7	4.83×10^{-3}	1.97×10^{-4}	106
Rio de Janeiro (Brazil)	29.2	307	35	1.05×10^{-2}	1.20×10^{-3}	107
Katowice (upper Silesia, Poland)	31	157	8.2	5.07×10^{-3}	2.65×10^{-4}	101
Porto Marghera (Italy)	31	200	9.3	6.45×10^{-3}	3.00×10^{-4}	108
Erzgebirge (Germany)	32.5	188	3	5.79×10^{-3}	9.23×10^{-5}	109
Barcelona (Spain)	35	260	52	7.43×10^{-3}	1.49×10^{-3}	110
Santa Catarina (Mexico)	36.15	466	16	1.29×10^{-2}	4.42×10^{-4}	111
Escobedo (Mexico)	37.78	493	13	1.31×10^{-2}	3.44×10^{-4}	111
Arnhem (the Netherlands)	38.95	241	13.5	6.19×10^{-3}	3.47×10^{-4}	112
Mira Loma (Southern California, average)	41.8	581	75	1.39×10^{-2}	1.79×10^{-3}	113
Taif (residential area, Saudi Arabia)	46	2000	5.3	4.35×10^{-2}	1.15×10^{-4}	114
Taif (industrial site, Saudi Arabia)	47	2300	13	4.89×10^{-2}	2.77×10^{-4}	114
Azusa (California)	47.1	281.9	13.4	5.99×10^{-3}	2.85×10^{-4}	103
Edison (New Jersey)	49.6	1953	10	3.94×10^{-2}	2.02×10^{-4}	115
Bursa (Turkey)	53	875	15	1.65×10^{-2}	2.83×10^{-4}	116
Karachi (summer, Pakistan)	55.89	3360	56	6.01×10^{-2}	1.00×10^{-3}	117
New Delhi (summer, high traffic, India)	58.2	710	20	1.22×10^{-2}	3.44×10^{-4}	118

Milan (Winter, Italy)	58.6	309	18	5.27×10^{-3}	3.07×10^{-4}	[104]
Thessaloniki (cold period, Greece)	60.9	2890	93	4.75×10^{-2}	1.53×10^{-3}	[119]
Thessaloniki (warm period, Greece)	70.6	4094	66	5.60×10^{-2}	9.35×10^{-4}	[119]
Córdoba City (Argentina)	70.87	325	8	4.59×10^{-3}	1.13×10^{-4}	[120]
Shanghai (China)	71.61	424.93	9.47	5.93×10^{-3}	1.32×10^{-4}	[121]
Yong'an (Winter, China)	79.01	582.1	19.6	7.37×10^{-3}	2.48×10^{-4}	[122]
Yong'an (Spring, China)	83.26	736.1	16.5	8.84×10^{-3}	1.98×10^{-4}	[122]
Karachi (winter, Pakistan)	98.44	3706	39	3.77×10^{-2}	3.96×10^{-4}	[117]
Ji'nan (urban site, China)	101	1040	30	1.03×10^{-2}	2.97×10^{-4}	[123]
Guangzhou (China)	104.58	66	60	6.31×10^{-4}	5.74×10^{-4}	[124]
Argra (urban, India)	104.9	1900	200	1.81×10^{-2}	1.91×10^{-3}	[125]
Pune (India)	113.8	2090	339	1.84×10^{-2}	2.98×10^{-3}	[126]
Beijing (Summer, China)	125	1060	44.7	8.48×10^{-3}	3.58×10^{-4}	[127]
Ji'nan (industrial site, China)	130	2410	40	1.85×10^{-2}	3.08×10^{-4}	[123]
Beijing (Winter, China)	138	1330	53.2	9.64×10^{-3}	3.86×10^{-4}	[127]
Beijing (China)	182.2	1180	70	6.48×10^{-3}	3.84×10^{-4}	[128]
New Delhi (winter, high traffic, India)	276.9	1150	70	4.15×10^{-3}	2.53×10^{-4}	[118]
Peat fire episode (Indonesia)	640	4810	100	7.52×10^{-3}	1.56×10^{-4}	[129]
Iron and copper medians	–	–	–	*8.14×10^{-3}*	*3.07×10^{-4}*	*This study*

Table S6. PM2.5 and SOA concentrations with mass fractions as quantified in PM2.5 collected at different sampling sites throughout the world.

**Sampling location**	**PM2.5 (μg m^{-3})**	**SOA (μg m^{-3})**	**SOA mass fraction**	**Reference**
Amazon (Brazil)	1.8	0.34	0.189	[130]
Hyytiälä (Finland)	2	1.2	0.60	[131]
Storm Peak (Colorado)	2.1	0.7	0.333	[131]

Jungfraujoch (Switzerland)	2.2	1.2	0.545	[131]
Duke Forest (North Carolina)	2.8	1.3	0.464	[131]
Chebogue Pt. (Canada)	2.9	1.5	0.517	[131]
Edinburgh (Scotland)	3	1.2	0.400	[131]
Mainz (Germany)	4.3	1.1	0.256	[131]
Boulder (Colorado)	4.4	2.5	0.568	[131]
Manchester (winter, UK)	5.2	0.6	0.115	[131]
Chelmsford (UK)	5.3	1.8	0.340	[131]
Vancouver (Canada)	7	2.5	0.357	[131]
Okinawa (Japan)	7.9	1.7	0.215	[131]
Off New England Coast	8.5	4.9	0.576	[131]
Thompson Farm (New Hampshire)	9.5	4.2	0.442	[131]
Zurich (winter, Switzerland)	9.6	4.3	0.448	[131]
Cheju (South Korea)	10.7	4	0.374	[131]
Fukue (Japan)	11	3.6	0.327	[131]
New York City (winter, New York)	11.6	2.6	0.224	[131]
New York City (summer, New York)	12.2	4.8	0.393	[131]
Pinnacle Park (New York)	12.3	5.4	0.439	[131]
Houston (Texas)	12.8	2.7	0.211	[131]
Tokyo (summer, Japan)	13.2	4.7	0.356	[131]
Manchester (summer, UK)	14.3	3	0.210	[131]
Pittsburgh (Pensylvenia)	14.7	3.1	0.211	[131]
Tokyo (winter, Japan)	16.2	2.3	0.142	[131]
Taunus (Germany)	16.3	7.9	0.485	[131]
Riverside (California)	19.1	7	0.366	[131]
Zurich (summer, Switzerland)	25.5	5.1	0.200	[131]
Mexico City (Mexico)	26.8	8.1	0.302	[131]

Guangzhou (China)	69.1	12.5	0.181	[132]
Beijing (China)	79.9	16.6	0.208	[132]
Shanghai (China)	90.7	11.1	0.122	[132]
Beijing (China)	158.5	40.9	0.258	[132]
Xi'an (China)	345.1	53.5	0.155	[132]
SOA median	–	–	*0.333*	*This study*

Table S7. PM2.5, Phenanthrenequinone (PQN), 1,4-naphthoquinone (1,4-NQN) and 1,2-naphthoquinone (1,2-NQN) concentrations with mass fractions as quantified in PM2.5 collected at different sampling sites throughout the world. *see SI section on particulate pollutant concentrations in the ELF.

Sampling location	PM2.5 (μg m^{-3})	PQN (ng m^{-3})	1,2-NQN (ng m^{-3})	1,4-NQN (ng m^{-3})	PQN mass fraction	1,2-NQN mass fraction	1,4-NQN mass fraction	Reference
Umea (Sweden)	7.8	Unknown	Unknown	0.03	2.6×10^{-6}	1.3×10^{-6}	1.3×10^{-6}	[133]
Athens (Greece)	35.6	0.071	0.157	0.26	2.69×10^{-6}	5.95×10^{-6}	9.86×10^{-6}	[134]
Mazar-e Sharif (Afghanistan)	69	Unknown	Unknown	0.027	1.06×10^{-6}	5.28×10^{-7}	5.28×10^{-7}	[133]
Kabul (Afghanistan)	86	Unknown	Unknown	0.2	6.28×10^{-6}	3.14×10^{-6}	3.14×10^{-6}	[133]
Atascadero (California)	5*	0.023	0.0127	0.0246	6.21×10^{-6}	3.43×10^{-6}	6.64×10^{-6}	[120]
Birmingham (UK)	15*	4.6	3.2	1.7	4.14×10^{-4}	2.88×10^{-4}	1.53×10^{-4}	[136]
Lake Elsinore (California)	20*	0.311	0.246	0.14	2.1×10^{-5}	1.66×10^{-5}	9.45×10^{-6}	[135]
Norfolk (UK)	5*	0.058	0.024	0.012	1.57×10^{-5}	6.48×10^{-6}	3.24×10^{-6}	[137]
Riverside (California)	25*	0.57	0.06	0.23	3.08×10^{-5}	3.24×10^{-6}	1.24×10^{-5}	[138]
Quinones median	–	–	–	–	*6.28* $\times 10^{-6}$	*3.43* $\times 10^{-6}$	*6.64* $\times 10^{-6}$	*This study*

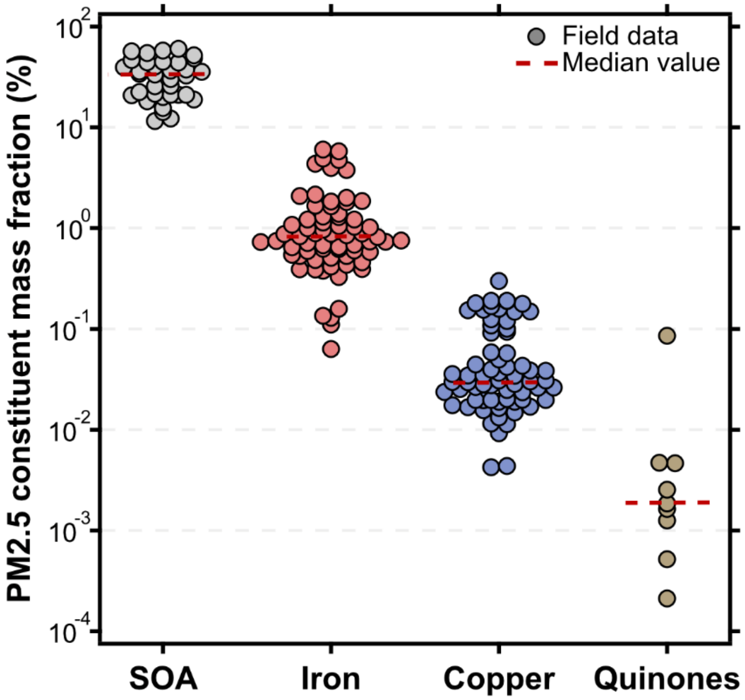

Figure S1. Mass fractions of all redox-active PM2.5 constituents quantified in field data (Tabs. S5-7). The median mass fraction of each redox-active PM2.5 constituent is indicated by the red horizontal line.

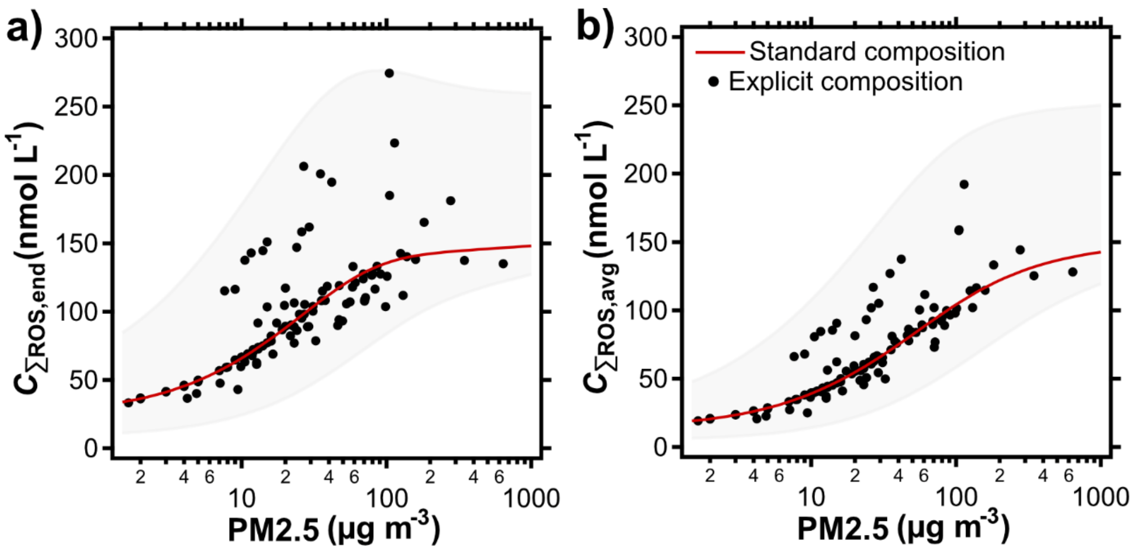

Figure S2. (a) ROS concentration, $C_{\Sigma ROS,end}$, after pollutant exposure, and (b) the average ROS concentration, $C_{\Sigma ROS,avg}$, during pollutant exposure as a function of PM2.5 concentration. The solid lines represent a standard composition of PM2.5 using median mass fractions of the redox-active constituents. Black markers represent calculations using explicit PM2.5 composition field data (Tab. S5-7)

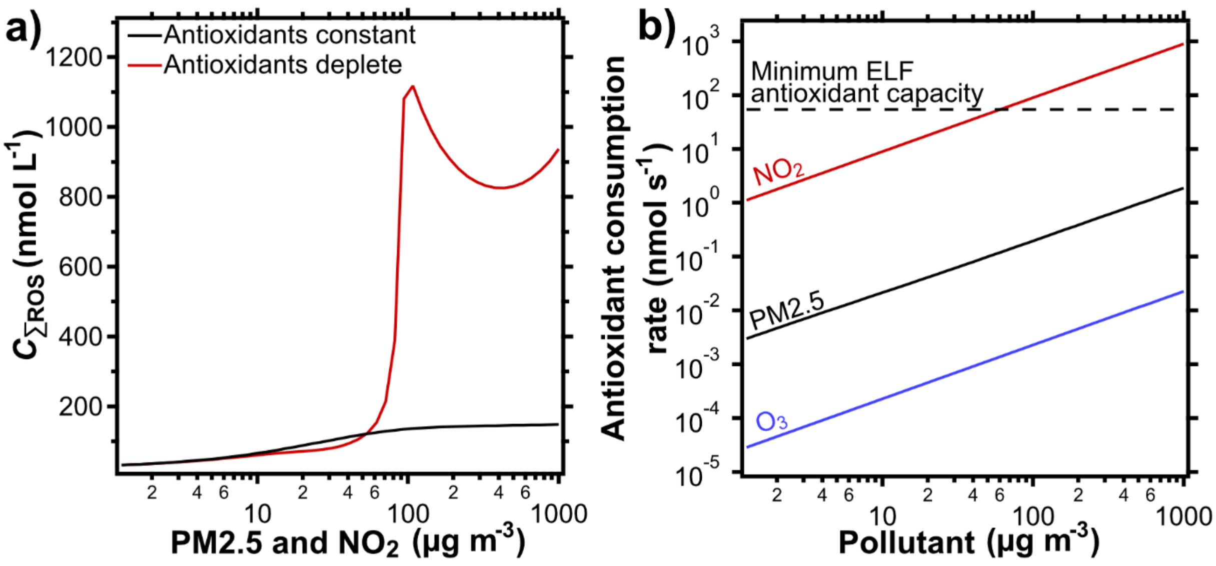

Figure S3. (a) ROS concentrations, $C_{\Sigma ROS}$, as a function of pollutant concentration with and without consideration of antioxidant depletion in the model. One µg of the co-pollutant NO_2 is added per µg PM2.5. For O_3, a constant concentration of 30 µg m^{-3} was used, independent of PM2.5 concentration. (b) Antioxidant consumption rate of NO_2, PM2.5 and O_3 as a function of pollutant concentration. The dashed line indicates the antioxidant consumption rate at which the ELF antioxidants would deplete in healthy humans within two hours of pollutant exposure without the consideration of enzymes or antioxidant replenishment.

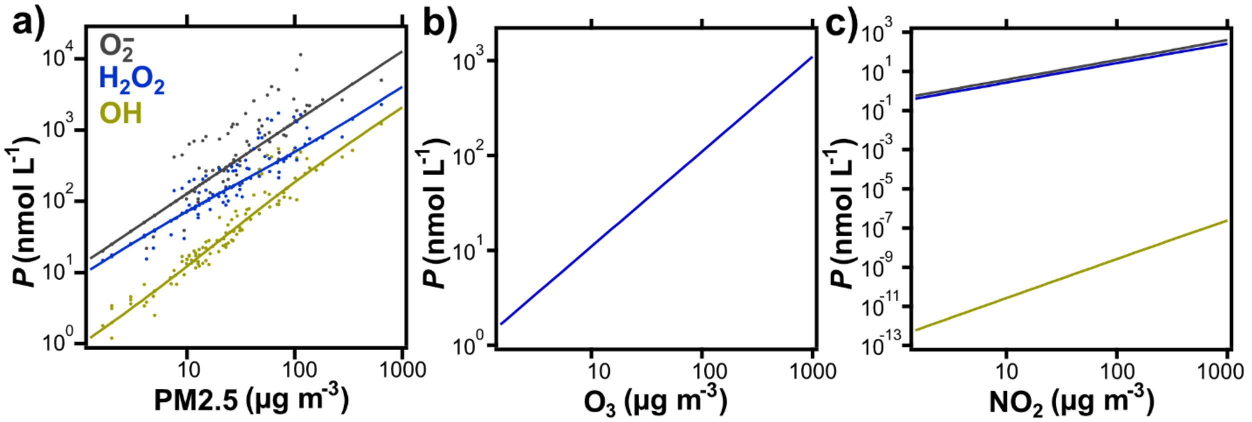

Figure S4. Gross chemical production of individual ROS in the ELF solely associated with and as a function of the concentration of three pollutants: (a) PM2.5, (b) O_3, and (c) NO_2. Simulations were carried out using only the single pollutant. In panel a, the solid lines represent standard PM2.5 composition, and the markers represent explicit PM2.5 composition data for redox-active constituents (Tabs. S5-7). O_3 does not contribute to production of O_2^- or OH in the model.

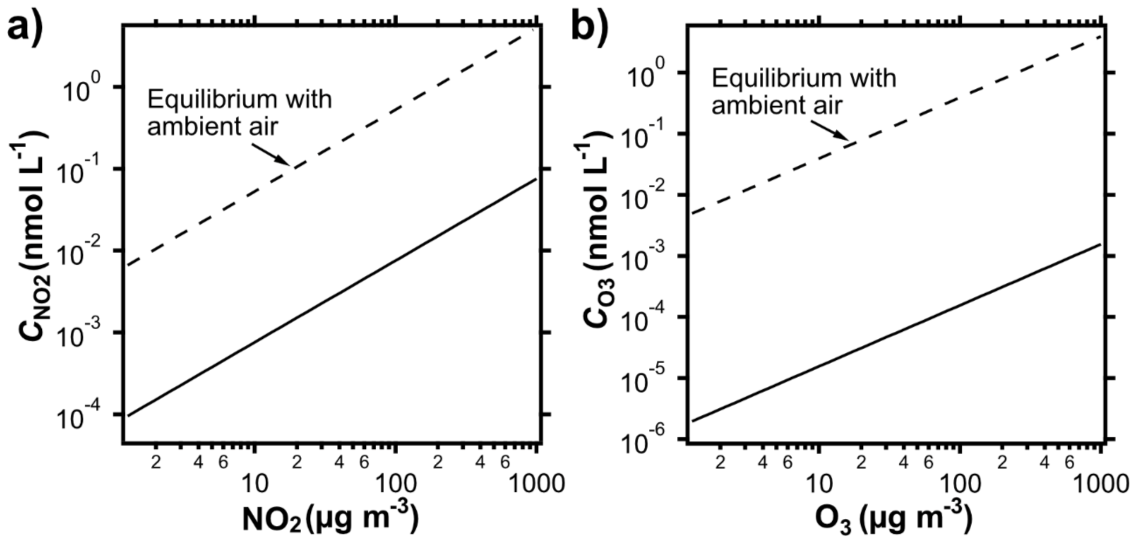

Figure S5. Aqueous-phase (a) NO_2 concentration and (b) O_3 concentration in the ELF as a function of pollutant concentration and comparison to hypothetical saturation with respect to ambient air. Dashed lines indicate saturation concentrations assuming Henry's law equilibrium, which are calculated using the ambient gas phase pollutant concentration and the dimensionless water–air partitioning coefficients for O_3 and NO_2, respectively (Table S2).

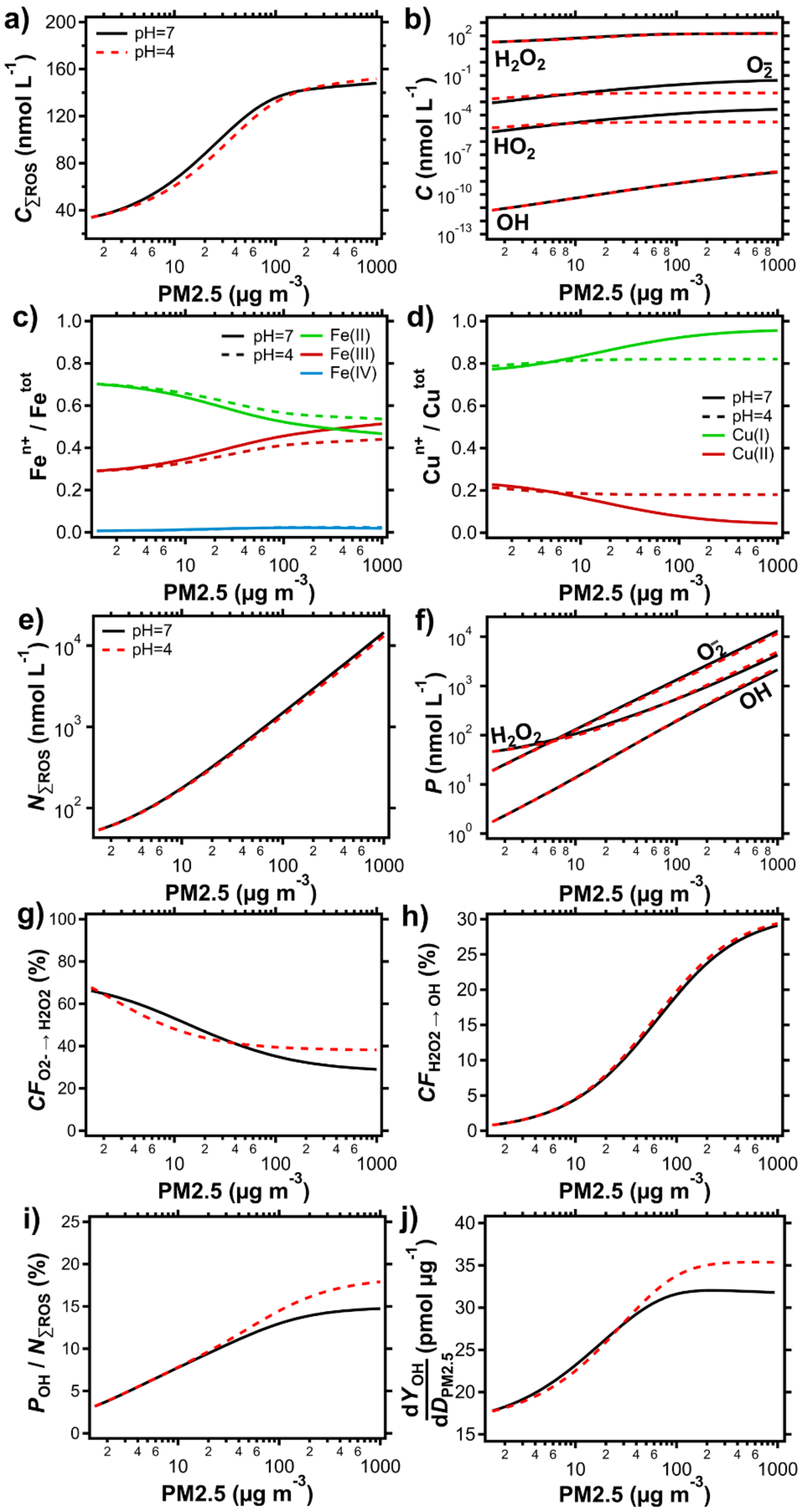

Figure S6. Effect of using different pH on the main results (panels a,b, e-j) and transition metal valence state (panels c,d) using the median mass fractions of all redox-active PM2.5 constituents, co-varied NO_2 (PM2.5:NO_2 = 1:1) and an O_3 concentration of 30 µg m^{-3}.
